# The Effect of Heat Stress and Dehydration on Carbohydrate Use During Endurance Exercise: A Systematic Review and Meta-Analysis

**DOI:** 10.1007/s40279-025-02294-3

**Published:** 2025-08-20

**Authors:** Loïs Mougin, Heather Z. Macrae, Lee Taylor, Lewis J. James, Stephen A. Mears

**Affiliations:** 1 School of Sport, Exercise and Health Sciences, Loughborough University, Loughborough, Leicestershire LE11 3TU UK; 2 School of Sport, Exercise and Rehabilitation, Faculty of Health, University of Technology Sydney, Sydney, Australia; 3 Human Performance Research Centre, University of Technology Sydney, Sydney, Australia

## Abstract

**Background:**

Carbohydrate metabolism during prolonged endurance exercise can be influenced by heat stress and dehydration. While heat exposure and dehydration have been shown to independently affect glycogen use and carbohydrate oxidation, their combined impact remains unclear. No previous review has systematically evaluated the effects of these factors on carbohydrate metabolism during prolonged endurance exercise or undertaken a meta-analysis.

**Objective:**

The aim was to systematically review the literature and meta-analyse the effects of heat stress (hot compared to temperate conditions) and dehydration (dehydrated compared to hydrated status) on (1) respiratory exchange ratio, (2) carbohydrate oxidation and (3) glycogen use.

**Methods:**

A Preferred Reporting Items for Systematic Reviews and Meta-Analyses (PRISMA)-compliant systematic review with meta-analysis was completed (https://osf.io/uq8n5). PubMed/MEDLINE and SportDiscus databases were searched for original articles (published up to November 2024) that assessed changes in (main outcomes) (1) respiratory exchange ratio, (2) carbohydrate oxidation or (3) glycogen use. The population included healthy, active, trained adults (> 18 years). Interventions involved exercise in hot conditions compared to temperate conditions and/or dehydration compared to a hydrated state. The exercise duration was required to be ≥ 15 min. Meta-analysis was performed using a random-effects model to calculate standardised mean differences (SMDs) between experimental conditions (hot compared to temperate conditions and/or dehydrated compared to hydrated statuses). Heterogeneity was assessed using *χ*^2^ and *I*^2^ statistics, with significance set at *P* ≤ 0.05.

**Results:**

Fifty-one studies (502 participants; 31 females) were included. Carbohydrate oxidation (SMD 0.29, *P* = 0.006) and glycogen use (SMD 0.78, *P* = 0.006) were greater in hot conditions compared to temperate conditions. In a dehydrated state, carbohydrate oxidation (SMD 0.31, *P* = 0.002) and glycogen use (SMD 0.62, *P* = 0.003) were greater compared to in a hydrated state. Greater carbohydrate oxidation in a dehydrated compared to a hydrated state was observed in hot (SMD 0.37, *P* = 0.001) but not in temperate conditions (SMD 0.27, *P* = 0.199).

**Conclusion:**

Carbohydrate utilisation increases during prolonged endurance exercise in hot conditions. Dehydration appears to increase carbohydrate use, especially when combined with heat stress; however, these effects are not consistently observed under temperate conditions. Consequently, dehydration does not appear to be the primary driver of elevated carbohydrate utilisation but may play a significant role by affecting thermoregulatory responses.

**Supplementary Information:**

The online version contains supplementary material available at 10.1007/s40279-025-02294-3.

## Key Points

**Table:** 

Carbohydrate use, including respiratory exchange ratio, carbohydrate oxidation, and glycogen utilisation, was consistently higher in hot conditions compared to temperate environments.
Dehydration increased carbohydrate oxidation, respiratory exchange ratio and glycogen use. However, when separating studies examining hydration state effects in hot versus temperate conditions, dehydration significantly increased carbohydrate oxidation and respiratory exchange ratio only in hot environments, with no significant differences observed in temperate conditions.
These findings suggest that heat exposure enhances carbohydrate utilisation during prolonged endurance exercise, while the effects of dehydration remain less clear, particularly under temperate conditions.

## Introduction

Exercise in hot environments is becoming increasingly common due to global warming (World Meteorological Organization 2023). Heat exposure, combined with metabolic heat production during exercise, triggers thermoregulatory responses, such as increased sweat production, to prevent/mitigate excessive elevations in body core temperature [1]. Increased evaporative heat loss is facilitated by an increased skin blood flow [2], leading to competing cardiovascular system demands: (1) supplying active skeletal muscles with oxygen to meet metabolic needs and (2) promoting heat dissipation by increasing cutaneous blood flow [2]. These concurrent demands increase cardiovascular strain, resulting in cardiovascular drift over time [3], and can alter blood flow and oxygen delivery at the muscle level, affecting substrate metabolism and glycogen use, potentially contributing to the performance impairment observed during prolonged exercise in hot compared to temperate environments [4]. Heat exposure has been shown to alter carbohydrate metabolism during submaximal exercise, as evidenced by increased muscle glycogen utilisation [5–7], elevated blood lactate accumulation [5, 6, 8–10] and higher whole-body carbohydrate oxidation [5, 6, 8, 11–13].

A common consequence of exercising in hot environments is dehydration, due to increased evaporative heat loss and inadequate fluid replacement [14, 15]. Dehydration amplifies cardiovascular drift observed in hot conditions by reducing plasma and blood volume, which restricts blood flow redistribution and impairs oxygen delivery to working muscles [14, 16]. These responses likely affect muscle metabolism and substrate utilisation [17, 18]. Recent studies, investigating the impact of hydration status, have reported that dehydration increases muscle glycogen use [18–21], and muscle lactate concentrations [17], even in the absence of heat exposure [17, 19–21]. However, the influence of dehydration on whole-body carbohydrate oxidation remains unclear, with some studies [17, 19, 21, 22], but not all [18, 20], reporting increased carbohydrate oxidation under a dehydrated hydration status.

To date, no systematic review and meta-analysis have examined the effects of heat stress on energy metabolism and substrate utilisation during prolonged exercise; however, two narrative reviews have attempted to describe these effects [23, 24]. A key limitation of these reviews is their restricted search strategies, as neither followed Preferred Reporting Items for Systematic Reviews and Meta-Analyses (PRISMA) guidelines nor implemented systematic methodologies or conducted meta-analyses. Moreover, these reviews included only studies reporting respiratory exchange ratio (RER) or direct substrate oxidation data, despite carbohydrate oxidation being calculable through indirect calorimetry using measured gas exchanges [25, 26]. Moreover, no reviews have addressed the effects of hydration status on carbohydrate use during prolonged exercise, nor differentiated between the impacts of elevated core temperature and dehydration on carbohydrate metabolism during exercise under heat stress.

Therefore, the aim of this systematic review, and associated meta-analyses, was to evaluate the effects of heat stress (hot compared to temperate conditions) and hydration (dehydrated compared to hydrated statuses) on carbohydrate metabolism (i.e. RER, carbohydrate oxidation and muscle glycogen use) during prolonged endurance exercise (≥ 15 min) in healthy, active, trained adults (> 18 years). It was hypothesised that both heat stress and dehydration would significantly increase reliance on carbohydrate metabolism during prolonged exercise, leading to increased muscle glycogen use and carbohydrate oxidation compared to exercise performed under temperate conditions and hydrated statuses.

## Methods

A pre-registered (Open Science Framework—https://osf.io/uq8n5) systematic review and meta-analysis was conducted in accordance with the updated PRISMA guidelines [27].

### Literature Search

A systematic review of the literature and meta-analyses were conducted on the effects of heat stress (hot compared to temperate conditions) and dehydration (dehydrated compared to hydrated status), during prolonged endurance exercise on (1) RER, (2) carbohydrate oxidation and (3) glycogen use. PubMed/MEDLINE and SportDiscus were independently searched, and articles published up to December 2023 were included, with the search updated in November 2024 to incorporate more recent studies. For both databases, the following terms were searched for in ‘all fields’: (carbohydrate OR CHO OR glucose OR fructose OR fat OR lipid OR substrate oxidation OR substrate use OR substrate utilisation OR substrate utilization OR glucose oxidation OR muscle metabolism OR RER OR Respiratory exchange ratio OR oxygen uptake OR oxygen consumption OR VO2* OR glyco*) AND (heat OR hot OR warm OR hydrat* OR dehydrat* OR hypohydrat* OR rehydrat* OR ambient temperature OR environmental temperature) AND (exercise OR endurance OR prolonged OR running OR cycling OR Rowing OR skiing OR walking) were searched only in ‘title/abstract’.

Analysis was restricted to articles in English and original research articles published in peer-reviewed journals. All years of publication were included.

**Fig. 1:**
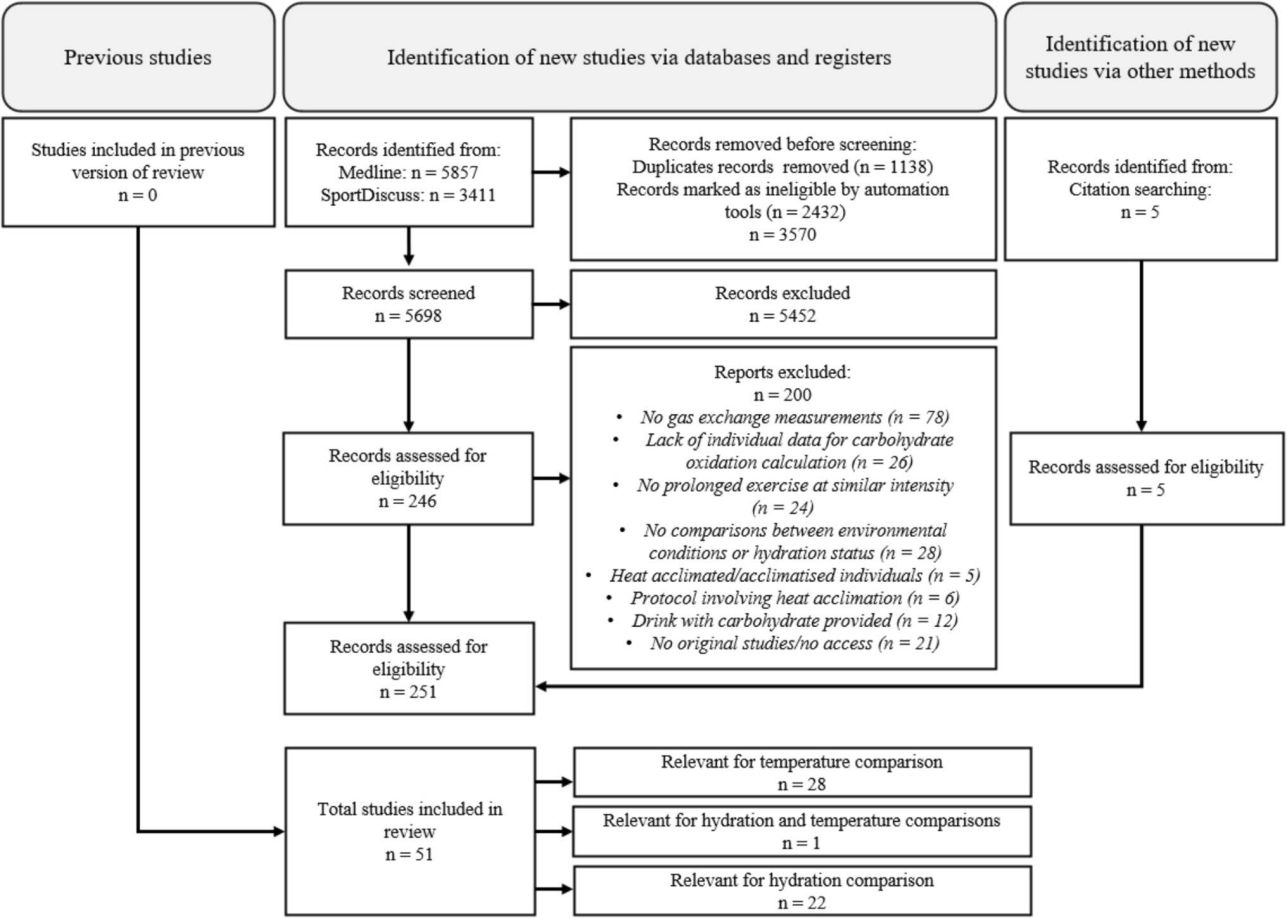
Overview of the selection process used for this systematic review

Reference lists from reviews [23, 24, 28] were also examined. Search results were exported into Covidence (Veritas Health Innovation, Melbourne, VIC, Australia) for screening and duplicate removal. Two authors (LM and HZM) independently screened the titles, followed by abstracts, for eligibility before retrieving relevant full-text articles based on the inclusion criteria (see below). At each stage, disagreements were resolved by discussion between LM and HZM, or by consulting other authors (SAM and LJJ). A flow diagram of the search strategy is presented in Fig. 1.

### Inclusion and Exclusion Criteria

To evaluate the effects of heat stress (hot compared to temperate conditions) and hydration (dehydrated compared to hydrated statuses) on carbohydrate metabolism during prolonged endurance exercise, the inclusion and exclusion criteria in Table 1 were considered.

**Table 1:** Inclusion and exclusion criteria used for the systematic search

Inclusion criteria	Exclusion criteria
1) Population: Healthy, active, trained, adult (> 18 years) participants 2) Studies with a crossover design with comparison of carbohydrate oxidation between a temperate vs. hot condition (≥ 28 ºC) or hydrated vs. dehydrated status or both 3) Study of carbohydrate oxidation or respiratory exchange ratio or glycogen use 4) Information about the environmental condition (i.e. temperature) 5) Exercise protocol clearly defined (time and intensity) 6) Identical intensity and nutritional intake between trials 7) Exercise duration ≥ 15 min 8) All years of publication	1) Participants with previous medical condition or obesity 2) Use of exercise trials with free chosen (self-paced) exercise intensity 3) Heat acclimated/acclimatised participants 4) Non-human subjects 5) Researchers unable to access full text or if studies provide insufficient information

### Study Quality Assessment

Study bias was determined using the van Rosendale risk of bias scale [29] and was assessed independently by two investigators (LM and HZM), with disagreements resolved by a third author (SAM). Excellent methodological quality was considered with a score of ≥ 60% (see Supplementary material 1, Tables 1 and 2, in the electronic supplementary material) based on the criteria as set out by previous studies [29, 30], but no studies were excluded on account of a poor study-quality assessment score. In environmental physiology studies, it is difficult to blind both participants and investigators to the environmental conditions. Therefore, questions regarding blinding were removed when determining risk of bias scores, but it must be noted that all studies may have had increased bias due to this inability to blind. For hydration studies, blinding is possible, so questions regarding blinding were considered.

**Table 2:** A summary of the participants, exercise characteristics, environmental conditions and hydration strategies of the studies included in the heat exposure meta-analysis (in chronological order)

Article	Sample size	Population	Exercise	Duration and intensity	Outcomes	Time point	Environmental condition	Core, rectal, oesophageal or tympanic temperature	Hydration strategy	BM change
Fink et al., 1975 [7]	6	Physically active males; 21–39 y	Cycling	3*15 min @ 70–85% $\dot{V}$O_2max_	Glycogen use	Exercise-averaged	T: 9 °C & 55% RH; H: 41 °C & 15% RH	T: + 1.0 °C; H: + 1.8 °C	Fluid provided to maintain BM at pre-exercise level	T: − 0.2% BM; H: − 0.1% BM
Young et al., 1985 [8]	13	Healthy males; 22 y	Cycling	30 min @ 70% $\dot{V}$O_2max_	RER, glycogen use	End of exercise (RER), exercise-averaged (glycogen use)	T: 21 °C & 30% RH; H: 49 °C & 20% RH	T: 38.0 ± 0.1 °C; H: 38.7 ± 0.1 °C*	Ad libitum	–
Dolny and Lemon, 1988 [44]	8	Males; 28 y	Cycling	90 min @ 65% $\dot{V}$O_2max_	RER	Exercise-averaged	T: 20 °C & 53% RH; H: 30 °C & 59% RH	T: 38.0 ± 0.2 °C; H: 38.2 ± 0.1 °C*	Ad libitum	–
Barnett and Maughan, 1993 [45]	5	Fit males; 26 y	Cycling	60 min @ 55% $\dot{V}$O_2max_	RER, CHOox	End of exercise	T: 22 °C & 67% RH; H: 35 °C & 60% RH	–	No fluid provided	T: − 0.8 ± 0.3 kg; H: − 1.3 ± 0.4 kg*
Snow et al., 1993 [46]	7	Active males; 24 y	Cycling	40 min @ 70% $\dot{V}$O_2peak_	RER	Exercise-averaged	T: 20 °C & 20% RH; H: 40 °C & 20% RH	T: 38.1 ± 0.2 °C; H: 38.7 ± 0.1 °C*	No fluid provided	–
Nielsen and Kroug, 1994a [47]	7	Elite male runners; 27 y	Running	60 min @ 75% $\dot{V}$O_2max_	RER	End of exercise	T: 18 °C & 50–60% RH; H: 32 °C & 50–60% RH	–	400 mL of distilled water in both conditions	T: − 1.1% BM; H: − 2.2% BM
Nielsen and Kroug, 1994b [47]	5	Endurance trained males; 27 y	Cycling	150 min @ 60% $\dot{V}$O_2max_	RER	End of exercise	T: 20 °C & 30–40% RH; H: 40 °C & 30–40% RH	–	400 mL of distilled water in both conditions	T: − 0 to − 0.1% BM; H: − 2.5% BM
Febbraio et al., 1994a [6]	13	Endurance trained males; 21 y	Cycling	40 min @ 70% $\dot{V}$O_2max_ (measured in H condition)	RER, CHOox, glycogen use	End of exercise	T: 20 °C & 20% RH; H: 40 °C & 20% RH	T: 38.7 ± 0.1 °C; H: 39.7 ± 0.1 °C*	–	–
Febbraio et al., 1994b [5]	12	Endurance trained males; 22 y	Cycling	40 min @ 70% $\dot{V}$O_2max_ (measured in H condition)	RER, CHOox, glycogen use	End of exercise	T: 20 °C & 20% RH; H: 40 °C & 20% RH	T: 38.6 ± 0.1 °C; H: 39.6 ± 0.1 °C*	–	–
Young et al., 1995 [39]	18	Healthy males; 2 groups of 9; 20 y	Cycling	60 min @ 60% $\dot{V}$O_2max_	Glycogen use	Post exercise	T: 20 °C; H: 35 °C (water immersion)	Group 1 T: 37.4 ± 0.2 °C; H: 38.3 ± 0.1 °C Group 2 T: 37.9 ± 0.3 °C; H: 38.5 ± 0.1 °C	–	–
Hargreaves et al., 1996 [10]	6	Endurance trained males; 20 y	Cycling	40 min @ 65% V̇O_2peak_	RER, CHOox	End of exercise	T: 20 °C & 50% RH; H: 40 °C & 50% RH	T: 38.2 ± 0.2 °C; H: 39.1 ± 0.2 °C*	–	T: − 0.7 ± 0.1 kg; H: − 1.2 ± 0.1 kg*
Galloway and Maughan, 1997 [48]	8	Healthy males; 25 y	Cycling	60 min @ 70% $\dot{V}$O_2max_	RER, CHOox	End of exercise	T: 21 °C & 70% RH; H: 31 °C & 70% RH	T: 39.3 °C; H: 39.6 °C*	No fluid provided	–
Cheuvront and Haymes, 2001 [33]	8	Female marathon runners; 37 y	Running	30 km @ best marathon race pace	RER, CHOox	End of exercise	T: 20 °C & 54% RH; H: 30 °C & 55% RH	T: 38.4 ± 0.1 °C; H: 39.2 ± 0.2 °C	Ad libitum	T: − 2.4 ± 0.6 kg; H: − 3.2 ± 0.8 kg*
Marino et al., 2001 [49]	9	Highly trained endurance male runners; 25 y	Running	30 min @ 70% of peak treadmill running speed	RER	End of exercise	T: 15 °C & 60% RH; H: 35 °C & 60% RH	T: 38.6 ± 0.1 °C; H: 38.5 ± 0.2 °C*	–	–
Nybo and Nielsen, 2001 [11]	8	Endurance trained males; 23 y	Cycling	60 min @ 57% $\dot{V}$O_2max_	RER, CHOox	Exhaustion (30–50 min)	T: 18 °C & 40% RH; H: 40 °C & 20% RH	T: 37.8 ± 0.1 °C; H: 40.0 ± 0.1 °C*	T: 0.3 ± 0.1 L; H: 0.8 ± 0.1 L of pre-warmed water	T: − 0.7 ± 0.1% BM; H: − 0.7 ± 0.2% BM
Jentjens et al., 2002 [50]	9	Trained males; 24 y	Cycling	90 min @ 55% $\dot{V}$O_2max_	RER, CHOox, glycogen use	Average 60–90 min	T: 16 °C & 60% RH; H: 35 °C & 27% RH	T: 38.3 ± 0.1 °C; H: 38.9 ± 0.2 °C*	Fluid provided every 15 min: 3 mL/kg BM	T: − 1.3 ± 0.1 kg; H: − 3.0 ± 0.2 kg*
Yamashita et al., 2005 [51]	6	Healthy males; 19 y	Cycling	120 min @ 60% $\dot{V}$O_2max_	RER	End of exercise	T: 20 °C & 60% RH; H: 30 °C & 60% RH	T: 38.3 ± 0.3 °C; H: 38.9 ± 0.7 °C	–	T: − 1.2 ± 0.2 kg; H: − 2.0 ± 0.9 kg
Hayashi et al., 2006 [12]	13	Healthy males; 25 y	Cycling	60 min @ 50% $\dot{V}$O_2peak_	RER, CHOox	End of exercise	T: 10 °C; H: 35 °C; H + : 45 °C (perfused suit)	T: 37.3 °C; H: 38.6 °C; H + : 39.2 °C*	No fluid provided	–
Hettinga et al., 2007 [52]	10	Well trained males; 24 y	Cycling	20 min @ 60% of power output @ $\dot{V}$O_2max_	RER	Exercise-averaged	T: 16 °C & 20% RH; H: 36 °C & 3% RH	T: 37.0 ± 0.1 °C; H: 37.4 ± 0.6 °C*	–	–
Shorten et al., 2009 [53]	11	Active males; 21 y	Running	40 min @ 70% $\dot{V}$O_2peak_	RER, CHOox	End of exercise	T: 25 °C & 30% RH; H: 36 °C & 30% RH	T: 36.1 ± 0.3 °C; H: 38.9 ± 0.3 °C*	–	T: − 0.7 ± 0.2 kg; H: − 0.7 ± 0.4 kg
Fernandez-Elias et al., 2015 [18]	7	Endurance trained males; 25 y	Cycling	40 min @ 75% $\dot{V}$O_2max_	CHOox, glycogen use	Exercise-averaged	T: 25 °C & 28% RH; H: 36 °C & 25% RH	T: 38.5 ± 0.4 °C; H: 39.2 ± 0.4 °C*	–	T: − 2.7 ± 0.3% BM; H: − 3.0 ± 0.2% BM
O'Hearn et al., 2016 [54]	8	Healthy males; 25 y	Running	30 min @ 50% $\dot{V}$O_2max_	CHOox	End of exercise	T: 23 °C & 35% RH; H: 42 °C & 24% RH	T: 37.6 °C; H: 39.0 °C*	–	T: − 0.8% BM; H: − 1% BM*
Collins et al., 2017 [55]	12	Recreationally trained males; 25 y	Cycling	60 min @ 60% *W*_max_	CHOox	Exercise-averaged	T: 20 °C & 60% RH; H: 33 °C & 60% RH	T: 37.9 ± 0.3 °C; H: 38 ± 0.3 °C*	500 mL in both conditions	–
Hoffman et al., 2018 [35]	10	Endurance trained (4 females/6 males); 31 y	Running	120 min @ 60% $\dot{V}$O_2max_	CHOox	End of exercise	T: 22 °C & 44% RH; H: 30 °C & 35% RH; H + : 35 °C & 26% RH	T: 38.4 ± 0.4 °C; H: 38.8 ± 0.6 °C; H + : 39.6 ± 0.7 °C*	Ad libitum	T: − 1.7 ± 0.7% BM; H: − 1.9 ± 0.7% BM; H + : − 1.7 ± 0.7% BM
Maunder et al., 2020 [56]	20	Endurance trained males; 37 y	Cycling	20 min @ VT1 (measured in H condition)	CHOox	End of exercise	T: 18 °C & 60% RH; H: 35 °C & 60% RH	Higher in H*	Ad libitum	–
Foster et al., 2023 [37]	10	Trained participants (1 female/9 males); 30 y	Cycling	60 min @ 6 W/kg	RER, CHOox	End of exercise	T: 25 °C & 23% RH; H: 40 °C & 22% RH	T: 37.9 ± 0.3 °C; H: 38.0 ± 0.3 °C	Ad libitum	–
Schoerberlein et al., 2023 [36]	9	Healthy participants (4 females/5 males); 27 y	Cycling	30 min @ 35% (15 min) & 52% (15 min) $\dot{V}$O_2max_	RER, CHOox	End of exercise	T: 22 °C & 35% RH; H: 39 °C & 38% RH	T: 37.7 ± 0.3 °C; H: 38.1 ± 0.3 °C*	–	T: − 0.5 ± 0.1% BM; H: − 1.6 ± 0.3% BM*
Mora-Rodriguez et al., 2024 [38]	9	Healthy participants (1 females/8 males); 31 y	Cycling	50 min @ 58% $\dot{V}$O_2max_	RER, CHOox, Glycogen use	Exercise-averaged	T: 21 °C; H: 33 °C	T: 36.6 ± 0.6 °C; H: 37.2 ± 0.3 °C*	–	T: − 0.4 ± 0.1 kg; H: − 0.6 ± 0.2 kg*
Rosbrook et al., 2024 [57]	10	Healthy males; 25 y	Walking	90 min @ 1.56 m/s with incline to elicit VT1	RER, CHOox	End of exercise	T: 20 °C & 40% RH; H: 37 °C & 20% RH	T: 38.3 ± 0.2 °C; H: 39.3 ± 0.2 °C*	–	T: − 0.9 ± 0.6% BM; H: − 1.7 ± 0.6% BM*

*BM* body mass, *CHOox* carbohydrate oxidation, *H* hot, *H+* Second hot condition, *RER* respiratory exchange ratio, *RH* relative humidity, *T* temperate, $\dot{V}$*O*_*2max*_ maximal oxygen consumption, $\dot{V}$*O*_*2peak*_ peak oxygen consumption, *VT* ventilatory threshold, *W*_*max*_ maximum workload, *y* years

*Significant difference was reported between environmental conditions

### Data Extraction

A search of electronic databases and a manual review of references from existing reviews [23, 24, 28] retrieved relevant studies (Fig. 1). Duplicates and articles automatically marked as ineligible (i.e. by the enabled machine learning models provided by Covidence) on Covidence were removed. A first screening was performed on titles and abstracts, followed by a full-text screening. Each study, conserved after both screenings, was read and coded for the following descriptive variables: sample size, exercise characteristics, environmental conditions, core temperature, hydration strategy, hydration status, RER, carbohydrate oxidation and glycogen use.

Data were extracted into Microsoft Excel independently by two investigators (LM and HZM). Data extracted included information on the methods (study design), participants (population, characteristics), exercise protocol (time, intensity, type of exercise), environment (temperature, humidity), hydration (amount of water provided, timing), core temperature, hydration status (body mass and plasma volume) and outcomes (RER, carbohydrate oxidation, glycogen use). Assimilated data endpoints from the selected papers were tabulated (Tables 2 and 3). Data were collected directly from tables or within the text of the selected studies where possible, by contacting the authors or using Graph digitizing software (DigitizeIt, Braunschweig, Germany) in studies where plots only were published. When gas exchange individual data were provided, RER and carbohydrate oxidation were calculated using Frayn (1983) equations [25].

**Table 3:** A summary of the participants, exercise characteristics, environmental conditions and hydration strategies of the studies included in the hydration status meta-analysis (in chronological order)

Article	Sample size	Population	Exercise	Duration and intensity	Outcomes	Timepoint	Environmental condition	Core, rectal, oesophageal or tympanic temperature	Hydration strategy	BM	Plasma volume
Walsh et al., 1994 [58]	6	Endurance trained males; 27 y	Cycling	60 min @ 70% $\dot{V}$O_2peak_	RER	End of exercise	32 °C & 60% RH	F: 38.4 ± 0.4 °C; NF: 38.2 ± 0.4 °C	F: 400 mL before exercise and 120 mL every 10 min; NF: no fluid provided	F: − 0.2% BM; NF: − 1.8% BM*	F: − 17.7 ± 10.3%; NF: − 18.4 ± 10.4%
González-Alonso et al., 1995 [16]	7	Endurance trained males; 28 y	Cycling	120 min @ 62% $\dot{V}$O_2max_	RER, CHOox	End of exercise	35 °C & 48% RH	F: 38.4 ± 0.1 °C; NF: 39.4 ± 0.1 °C*	F: water to replace sweat loss; NF: 200 mL of fluid provided	F: − 0.5 ± 0.1% BM; NF: − 4.9 ± 0.2% BM*	F: − 6.4 ± 2.4%; NF: − 12.6 ± 0.5%*
Fallowfield et al., 1996 [34]	8	Healthy subjects (4 females/4 males); 21 y	Running	120 min @ 70% $\dot{V}$O_2max_	RER	75–120 min of exercise	20 °C	–	F: a bolus equivalent to 3 mL of water/kg BM followed by 2 mL of water/kg BM every 15 min; NF: no fluid provided	–	–
Hargreaves et al., 1996 [17]	5	Trained males; 27 y	Cycling	120 min @ 67% $\dot{V}$O_2peak_	RER, glycogen use	Exercise-averaged	20–22 °C	F: 38.0 ± 0.2 °C; NF: 38.6 ± 0.2 °C*	F: ~ 33 mL of water/kg BM; NF: no fluid provided	F: + 0.2 ± 0.1% BM; NF: − 4.9 ± 0.2% BM*	F: − 13.7 ± 2.8%; NF: − 24.3 ± 3.1%*
González-Alonso et al., 1997 [15]	7	Endurance trained males; 25 y	Cycling	30 min @ 72% $\dot{V}$O_2max_	RER, CHOox	End of exercise	35 °C & 50% RH	F: 39.3 ± 0.1 °C; NF: 39.3 ± 0.1 °C	F: water to replace BM loss; NF: dehydration prior exercise	F: -0.1 ± 0.2% BM; NF: − 4.4 ± 0.2% BM*	F: 2913 ± 53 mL; NF: 2756 ± 49 mL*
González-Alonso et al., 1997 [15]	8	Endurance trained males; 24 y	Cycling	30 min @ 72% $\dot{V}$O_2max_	RER, CHOox	End of exercise	2 °C	F: 38.1 ± 0.1 °C; NF: 38.2 ± 0.1 °C	F: water to replace BM loss; NF: dehydration prior exercise	F: − 0.0 ± 0.1% BM; NF: − 4.1 ± 0.1% BM*	F: 3035 ± 62 mL; NF: 2884 ± 73 mL*
González-Alonso et al., 1999 [9]	7	Endurance trained males; 27 y	Cycling	122–148 min @ 61% $\dot{V}$O_2max_	RER, CHOox	Exhaustion (122–148 min)	35 °C & 40–50% RH	No difference	F: large amount of fluid provided (4.3 L); NF: small amount of fluid provided (0.8 L)	F: − 0.0 ± 0.0% BM; NF: − 3.9 ± 0.3% BM*	–
Casa et al., 2000 [40]	8	Endurance trained males; 24 y	Cycling	15 min @ 70% $\dot{V}$O_2peak_	RER	End of exercise	36 °C	OF: 37.4 °C; IF: 37.9 °C; NF: 38.3 °C;* between NF & F	Dehydration (− 4% BM); then F: fluid provided equal to 50% of prior dehydration (OF & IF); NF: no fluid provided	Pre-exercise OF: − 2.0 ± 0.3% BM; IF: − 1.9 ± 0.3% BM; NF: − 3.9 ± 0.3% BM	OF: − 12.8%; IF: − 10.6%; NF: − 14.7%;* between NF & IF
Fritzsche et al., 2000 [59]	8	Endurance trained males; 22 y	Cycling	122 min @ 62% $\dot{V}$O_2max_	CHOox	End of exercise	35 °C & 43% RH	F: 38.5 ± 0.1 °C; NF: 39.2 °C	F: 3.28 ± 0.21 L of water provided; NF: 0.37 ± 0.02 L	F: − 1.0 ± 0.2% BM; NF: − 4.2 ± 0.2% BM*	F: − 6.6 ± 0.7%; NF: − 11.3 ± 0.8%*
Roy et al., 2000 [42]	10	Healthy males; 20 y	Cycling	60 min @ 61% $\dot{V}$O_2max_	RER, CHOox, glycogen use	End of exercise	22–24 °C & 35–45% RH	–	4 days diuretic induced hypohydration; ad libitum during exercise	–	F: 0.0 ± 0.0%; NF: − 14.6 ± 3.3%
Vallier et al., 2005 [60]	8	Trained males; 31 y	Cycling	180 min @ 60% $\dot{V}$O_2max_	RER	End of exercise	20–21 °C & 50% RH	F: 37.6 ± 0.3 °C; NF: 37.9 ± 0.3 °C	F: 400 mL before exercise and 200 mL every 20 min; NF: no fluid provided	F: − 2.2% BM; NF: − 4.1% BM*	–
Ebert et al., 2007 [61]	8	Well trained males; 28 y	Cycling	120 min @ 53% MAP	RER	Exercise-averaged	29 °C & 37% RH	F: 38.3 ± 0.2 °C; NF: 38.9 ± 0.2 °C*	F: 2.4 L of water provided; NF: 0.4 L	F: + 0.3 ± 0.4% BM; NF: − 2.5 ± 0.5% BM*	Significantly lower in NF
Del Coso et al., 2008 [62]	7	Endurance trained males; 27 y	Cycling	120 min @ 63% $\dot{V}$O_2peak_	RER, CHOox	End of exercise	35 °C & 27% RH	F: 38.7 ± 0.5 °C; NF: 39.4 ± 0.5 °C*	F: replace 97% loss; NF: no water provided	F: − 0.8% BM; NF: − 3.8% BM*	–
Merry et al., 2010 [41]	12	6 trained males; 31 y & 6 untrained males; 25 y	Cycling	40 min @ 70% $\dot{V}$O_2peak_	RER	End of exercise	24 °C & 50% RH	Trained: F: 37.4 ± 0.3 °C; NF: 37.6 ± 0.3 °C; Untrained: F: 37.3 ± 0.2 °C; NF: 37.6 ± 0.3 °C	F: replace 100% loss; NF: pre-hypohydration − 1.5–2% BM and replace 20% loss	Pre-exercise F: 0% BM; NF: − 1.5 to − 2% BM*	–
Gagnon et al., 2012 [63]	16	8 untrained males; and 8 trained males; 23 y & 27 y	Cycling	120 min @ 120 W	RER, CHOox	End of exercise	42 °C & 20% RH	Untrained F: 38.3 ± 0.2 °C; untrained NF: 38.7 ± 0.1 °C; trained F: 38.3 ± 0.2 °C; trained NF: 39.2 ± 0.2 °C	F: 400–700 mL every 30 min; NF: no fluid provided	Untrained F: − 0.6 ± 0.8% BM; untrained NF: − 2.3 ± 0.8% BM; trained F: − 0.4 ± 0.6% BM; trained NF: − 2.6 ± 1.0% BM;* between F; NF	–
Kelly et al., 2012 [64]	10	Physically active males; 21 y	Running	45 min @70% $\dot{V}$O_2peak_	RER, CHOox	End of exercise	19–20 °C & 45–50% RH	No difference	F: 500 mL before exercise & water provided during exercise to compensate 1.5 times sweat loss; NF: no fluid provided	F: − 0.3 ± 0.3% BM; NF: − 2.3 ± 0.4% BM*	–
Logan-Sprenger et al., 2012 [19]	9	Recreationally active females; 22 y	Cycling	120 min @ 65% $\dot{V}$O_2max_	RER, CHOox, glycogen use	End of exercise (RER), exercise-averaged (CHOox, glycogen use)	23 °C & 30% RH	F: 38.5 ± 0.2 °C; NF: 39.1 ± 0.2 °C*	F: water to replace BM loss; NF: no fluid provided	F: 0.0 ± 0.0% BM; NF: − 2.0 ± 0.2% BM*	F: − 4.1 ± 1.2%; NF: − 9.4 ± 1.5%
Logan-Sprenger et al., 2013 [20]	9	Active males; 22 y	Cycling	120 min @ 65% $\dot{V}$O_2max_	RER, CHOox, glycogen use	End of exercise (RER), exercise-averaged (CHOox, glycogen use)	23 °C & 32% RH	F: 38.2 ± 0.2 °C; NF: 38.7 ± 0.2 °C*	F: water to replace BM loss; NF: no fluid provided	F: 0.0 ± 0.0% BM; NF: − 2.7 ± 0.2% BM*	–
Fernandez-Elias et al., 2015 [18]	7	Endurance trained males; 25 y	Cycling	40 min @ 75% $\dot{V}$O_2max_	CHOox, glycogen use	Exercise-averaged	25 °C & 28% RH	T: 38.5 ± 0.4 °C; H: 39.2 ± 0.5 °C*	For all sessions: 150 min cycling in H environment to induce dehydration (− 4.6 ± 0.3% BM); F: water to replace BM loss; NF: no fluid provided	F: − 2.7 ± 0.3% BM; NF: − 5.6 ± 0.5% BM	F: − 15.9 ± 3.3%; NF: − 15.5 ± 5.8%
Logan-Sprenger et al., 2015 [21]	9	Trained males; 22 y	Cycling	90 min @ 65% $\dot{V}$O_2max_	RER, CHOox, glycogen use	End of exercise (RER), exercise-averaged (CHOox, glycogen use)	23 °C & 32% RH	F: 38.0 ± 0.5 °C; NF: 38.9 ± 0.7 °C*	F: water to replace BM loss; NF: no fluid provided	F: 0.0% BM; NF: − 2.3% BM*	–
James et al., 2017 [43]	7	Active males; 25 y	Cycling	8*15 min @ 50% PPO	RER, CHOox	End of exercise	34 °C & 50% RH	F: 38.2 ± 0.3 °C; NF: 38.4 ± 0.4 °C	For all sessions: 0.2 mL/kg BM of water provided every 10 min; F: additional water to replace BM loss via a gastric feeding tube; NF: no additional water provided	F: − 0.1 ± 0.1% BM; NF: − 2.4 ± 0.2% BM*	F: − 7.2 ± 2.9%; NF: − 12.3 ± 2.3%*
Funnell et al., 2019 [22]	14	Trained males; 25 y (2 groups: B & UB)	Cycling	120 min @ 50% *W*_peak_	RER, CHOox	End of exercise	31 °C & 48% RH	UB F: 37.9 ± 0.3 °C; UB NF: 38.5 ± 0.4 °C; B F: 38.0 ± 0.4 °C; B NF: 38.5 ± 0.5 °C	All sessions: 0.2 mL/kg BM; F: water to compensate sweat loss; NF: no fluid provided; B: nasogastric tube to provide fluid & UB: oral ingestion	UB F: − 0.6 ± 0.5% BM; UB NF: − 3.0 ± 0.5% BM; B F: − 0.5 ± 0.3% BM; B NF: − 3.0 ± 0.3% BM	UB F: − 9.3 ± 4.5%; UB NF: − 13.4 ± 0.5%; B F: − 10.6 ± 3.4%; B NF: − 11.9 ± 4.1%;* between F and NF
Campa et al., 2020 [65]	10	Active males; 23 y	Cycling	60 min @ 65% $\dot{V}$O_2max_	RER	Exercise-averaged	21 °C & 52% RH	–	F: 250 mL every 15 min; NF: no fluid provided	F: − 0.3 ± 0.3% BM; NF: − 1.8 ± 0.4% BM*	–
Funnell et al., 2023 [66]	17	Males intermittent game players; 22 y	Running	12*6 min @ 65% $\dot{V}$O_2max_	CHOox	End of exercise	23.9 °C & 44% RH	–	F: water to compensate 95% sweat loss (1622 ± 343 mL); NF: small amount of fluid provided (60 mL)	F: − 0.5 ± 0.3% BM; NF: − 2.2 ± 0.4% BM*	F: − 1.2 ± 2.3%; NF: − 4.5 ± 3.2%*

*B* blinded, *BM* body mass, *CHOox* carbohydrate oxidation, *F* fluid provided/hydrated condition, *H* hot, *IF* intravenous rehydration, *MAP* maximal aerobic power, *NF* no fluid provided/dehydrated condition, *OF* oral rehydration, *PPO* peak power output, *RER* respiratory exchange ratio, *RH* relative humidity,* T* temperate, *UB* unblinded, $\dot{V}$*O*_*2max*_ maximal oxygen consumption, $\dot{V}$*O*_*2peak*_ peak oxygen consumption, *W*_*peak*_ peak workload, *y* years

*Significant difference was reported between hydration status

### Data Analysis

Meta-analysis was conducted using RStudio (2023.03.0, RStudio, PBC, Boston, MA, USA) in order to aggregate, via a random-effects model [31], the standardised mean difference (SMD) in RER, carbohydrate oxidation and glycogen use, between temperate versus hot environment, or hydrated versus dehydrated status. Use of the SMD summary statistics allowed all effect sizes to be transformed into a uniform scale, which was then interpreted according to Hedges’ *g* criteria [32] allowing correction for small sample size, with SMDs of < 0.2, 0.2–0.5, 0.5–0.8 and > 0.8 representing *trivial*, *small*, *medium* and *large* effect sizes, respectively. Heterogeneity was assessed with the *χ*^2^ and *I*^2^ statistic; *P* > 0.10 indicates significant heterogeneity, and interpreted as follows: < 25% indicates low risk, 25–75% indicates moderate risk and > 75% indicates a high risk [31]. A *P* value ≤ 0.05 was considered statistically significant.

## Results

### Study Characteristics, Publication and Risks of Bias

The search across the two databases and citation searches produced a total of 9273 results. Duplicates and articles automatically marked as ineligible on Covidence resulted in 3570 articles removed. After screening titles and abstracts, 5452 articles were excluded. Of the 251 full-text articles screened, 51 studies were included in the final review (see Fig. 1 for specifics) including a total of 502 participants (31 females [6%]). Two studies included females only [19, 33], and five included both males and females [34–38].

Physiological responses in hot versus temperate conditions were compared in 29 studies (57%). Of the 23 studies (45%) comparing physiological responses in hydrated versus dehydrated status, 11 (48%) of them were performed in hot conditions (34.9 ± 3.9 °C and 36.3 ± 10.7% relative humidity [RH]), while 13 (57%) were performed in temperate conditions (i.e. 20.6 ± 5.9 °C and 40.3 ± 9.2% RH; one study was performed in hot and temperate conditions [15]). One study compared physiological responses in hot versus temperate conditions, and in hydrated versus dehydrated status [18].

Excellent methodological quality (i.e. a score of ≥ 60% based on criteria detailed in the ‘Methods’ section) was reached in 26 (out of 29) studies for temperature comparisons (Supplementary material 1, Table 1; see the electronic supplementary material) and in 17 (out of 23) studies for hydration comparisons (Supplementary material 1, Table 2). The order of the trials was randomised in 42 studies and non-randomised (or not indicated) in nine studies. Sensitivity analysis was conducted using studies with excellent methodological quality (i.e. score ≥ 60%) and randomised trial order (Supplementary Material 2), and the results were consistent with those presented below. Funnel plots were used to assess publication bias (Supplementary material 3, Fig. 1), and their symmetrical distribution, with most studies falling within the pseudo 95% confidence interval (CI), suggested a low risk of publication bias. For within-participant measurements, correlation coefficients were analysed in studies where individual data have been provided (*N* = 19), with *r* = 0.50 ± 0.27 for RER and *r* = 0.54 ± 0.28 for carbohydrate oxidation.

Regarding studies comparing physiological responses in temperate versus hot conditions, 27 studies (93%) involved exercising in environmental chambers with mean temperatures of 36.1 ± 4.6 °C and 38.0 ± 18.9% RH for hot conditions, and 19.8 ± 3.5 °C with 43.4 ± 16.6% RH for temperate conditions. One study was performed using a perfused suit [12], and one was performed using water immersion [39]. These studies were mainly performed using cycling (22 studies; 76%), running (six studies; 21%) or walking on an inclined treadmill (one study; 3%), with intensity varying from 35 to 85% maximal oxygen consumption ($\dot{V}$O_2max_). Among these 29 studies, 25 reported core temperature measurements, showing an increase of 0.84 ± 0.64 °C in hot conditions compared to temperate conditions.

In studies comparing between hydration statuses, dehydration was induced mostly by prolonged exercise with progressive fluid losses (18 studies; 78%). In these studies, a hydrated state was achieved through fluid intake to replace some/all of sweat losses. In five studies [15, 18, 40, 41] (one study included two substudies), a prior period of exercise was used to dehydrate, before participants either rehydrated or remained in a state of hypohydration so that at the start of the main experimental trial they were in different states of hydration. One study [42] used a diuretic in the 4 days before the prolonged exercise to induce hypohydration. In two studies [22, 43], participants were blinded regarding their hydration status. In all trials, the average body mass loss at the end of exercise was − 2.7 ± 1.1% of initial body mass in the dehydrated condition, compared to − 0.5 ± 0.8% in the hydrated condition. These studies mainly used cycling (20 studies; 87%) or running (three studies; 13%), with a varied intensity of 50–75% $\dot{V}$O_2max_.

### The Effect of Prolonged Exercise in Hot Versus Temperate Conditions on RER, Carbohydrate Oxidation and Glycogen Use

**Fig. 2:**
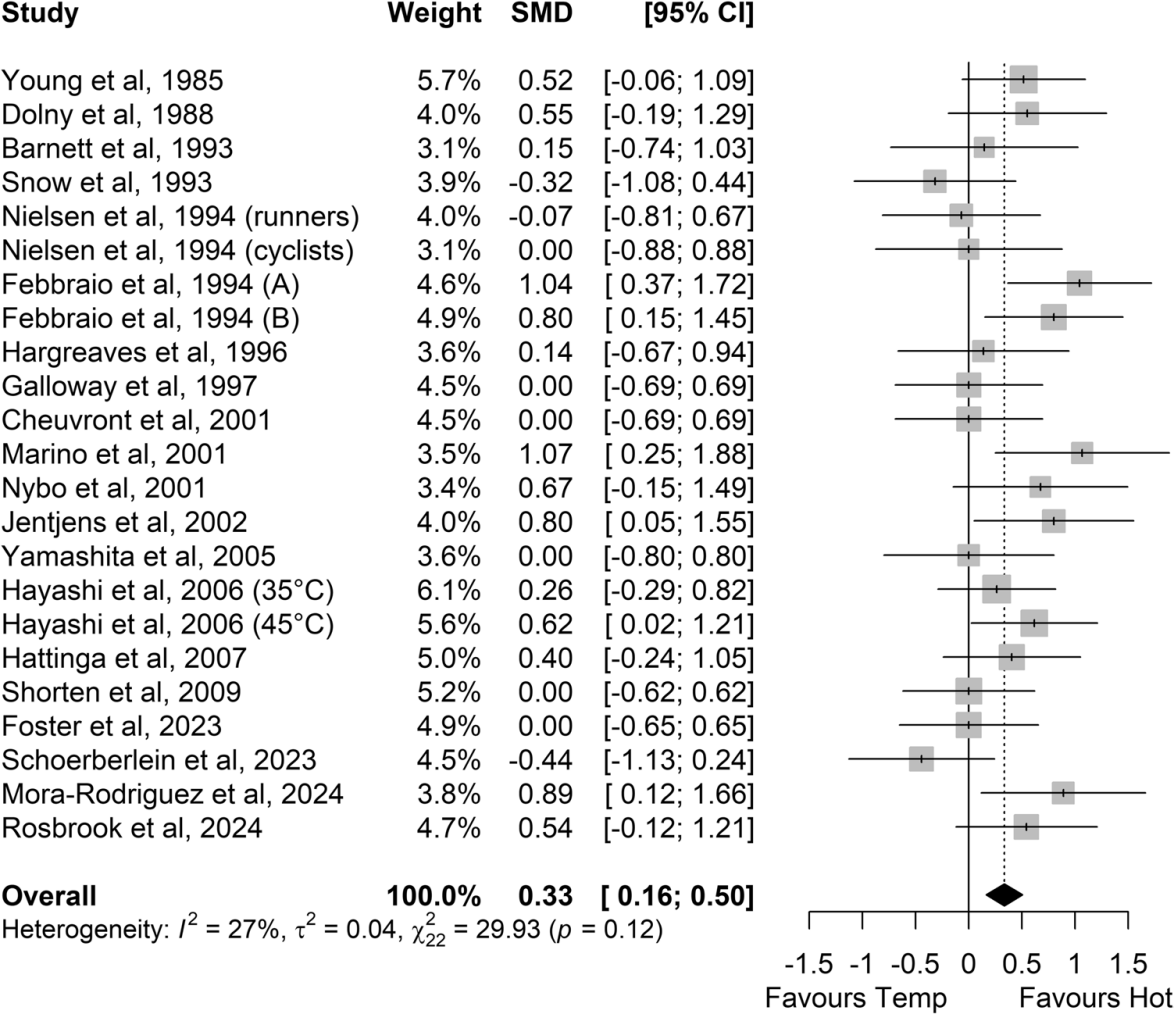
Comparison of the effects of prolonged exercise in hot vs. temperate (Temp) conditions on the respiratory exchange ratio. Forest plot shows standardised mean differences (SMDs) with 95% confidence intervals (CIs). Squares represent the SMD for each study. The diamond represents the pooled SMD for all studies

**Fig. 3:**
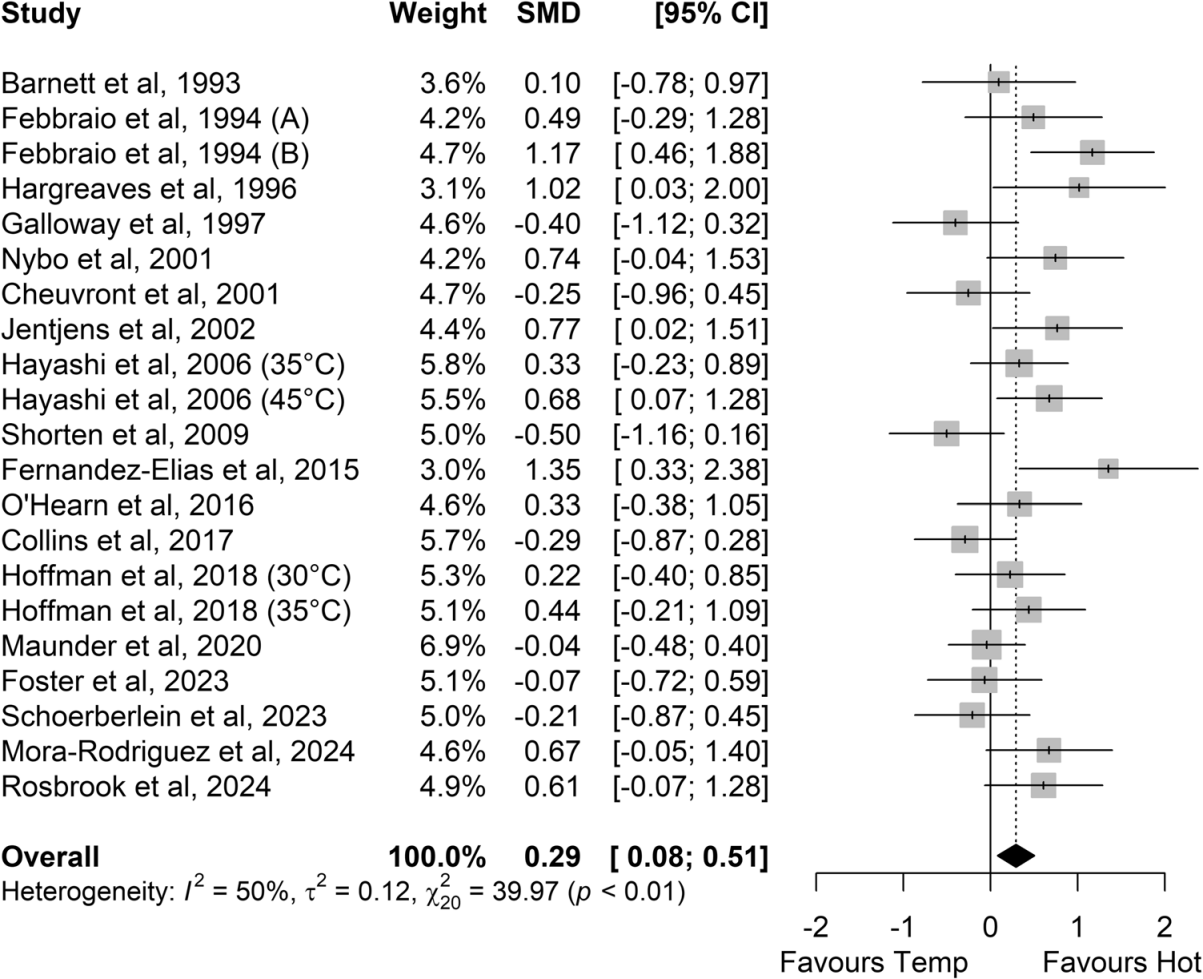
Comparison of the effects of prolonged exercise in hot vs. temperate (Temp) conditions on carbohydrate oxidation. Forest plot shows standardised mean differences (SMDs) with 95% confidence intervals (CIs). Squares represent the SMD for each study. The diamond represents the pooled SMD for all studies

**Fig. 4:**
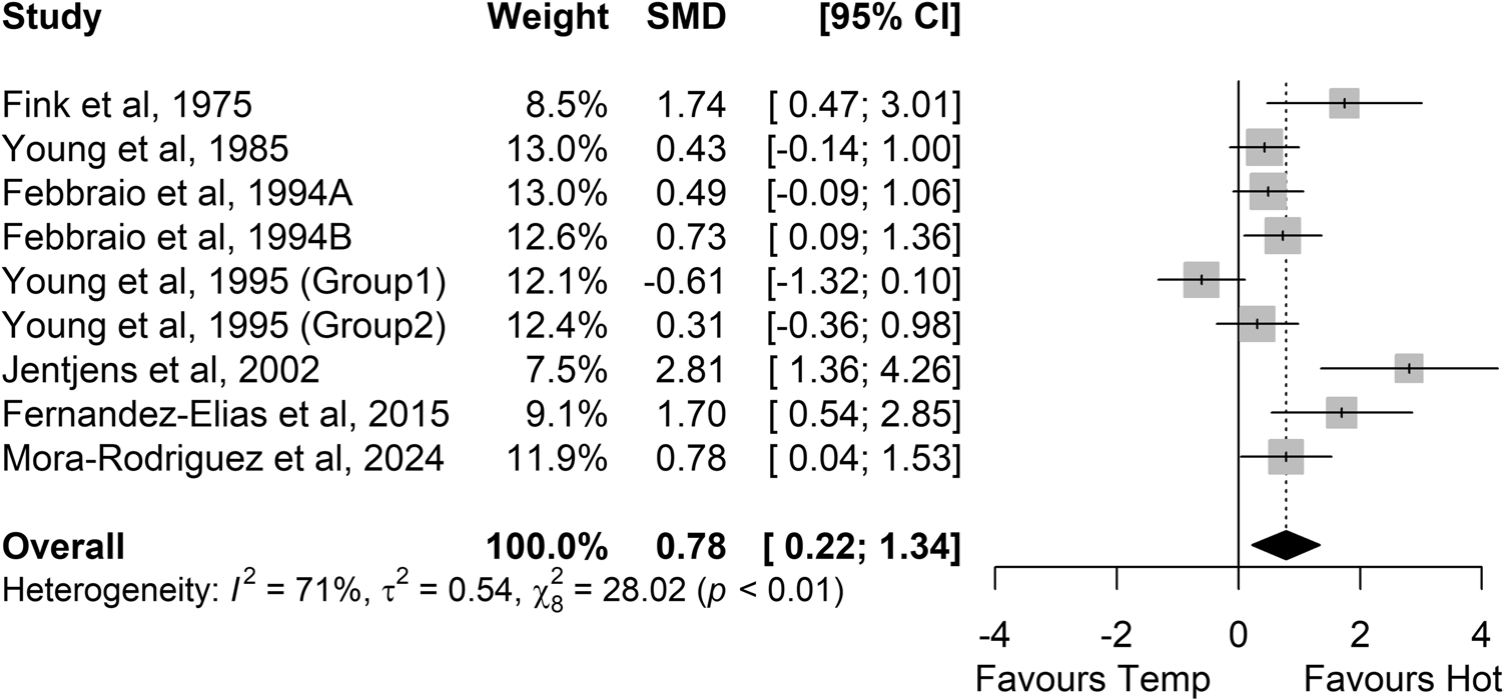
Comparison of the effects of prolonged exercise in hot vs. temperate (Temp) conditions on glycogen use. Forest plot shows standardised mean differences (SMDs) with 95% confidence intervals (CIs). *Squares* represent the SMD for each study. The diamond represents the pooled SMD for all studies

Greater RER values were found in hot conditions versus temperate conditions (SMD 0.33, 95% CI 0.16–0.50, *P* < 0.001; *Z* = 3.85, *small* effect; Fig. 2). In hot conditions, carbohydrate oxidation (SMD 0.29, 95% CI 0.08–0.51; *small* effect; *P* = 0.006; *Z* = 2.72; Fig. 3) and glycogen use (SMD 0.78, 95% CI 0.22–1.34; *medium* effect; *P* = 0.006; *Z* = 2.74; Fig. 4) were higher. Sixteen of the 29 studies comparing carbohydrate oxidation in temperate versus hot conditions reported changes in body mass, with five maintaining similar hydration levels by the end of exercise. Among these five studies, three (out of four) reported higher carbohydrate oxidation in hot conditions, while one study (out of one) observed increased glycogen use in the heat. Significant heterogeneity was detected among studies for carbohydrate oxidation (*I*^2^ = 50%; *P* < 0.01) and glycogen use (*I*^2^ = 71%; *P* < 0.01) with a moderate risk, but not for RER (*I*^2^ = 27%; *P* = 0.12).

**Fig. 5:**
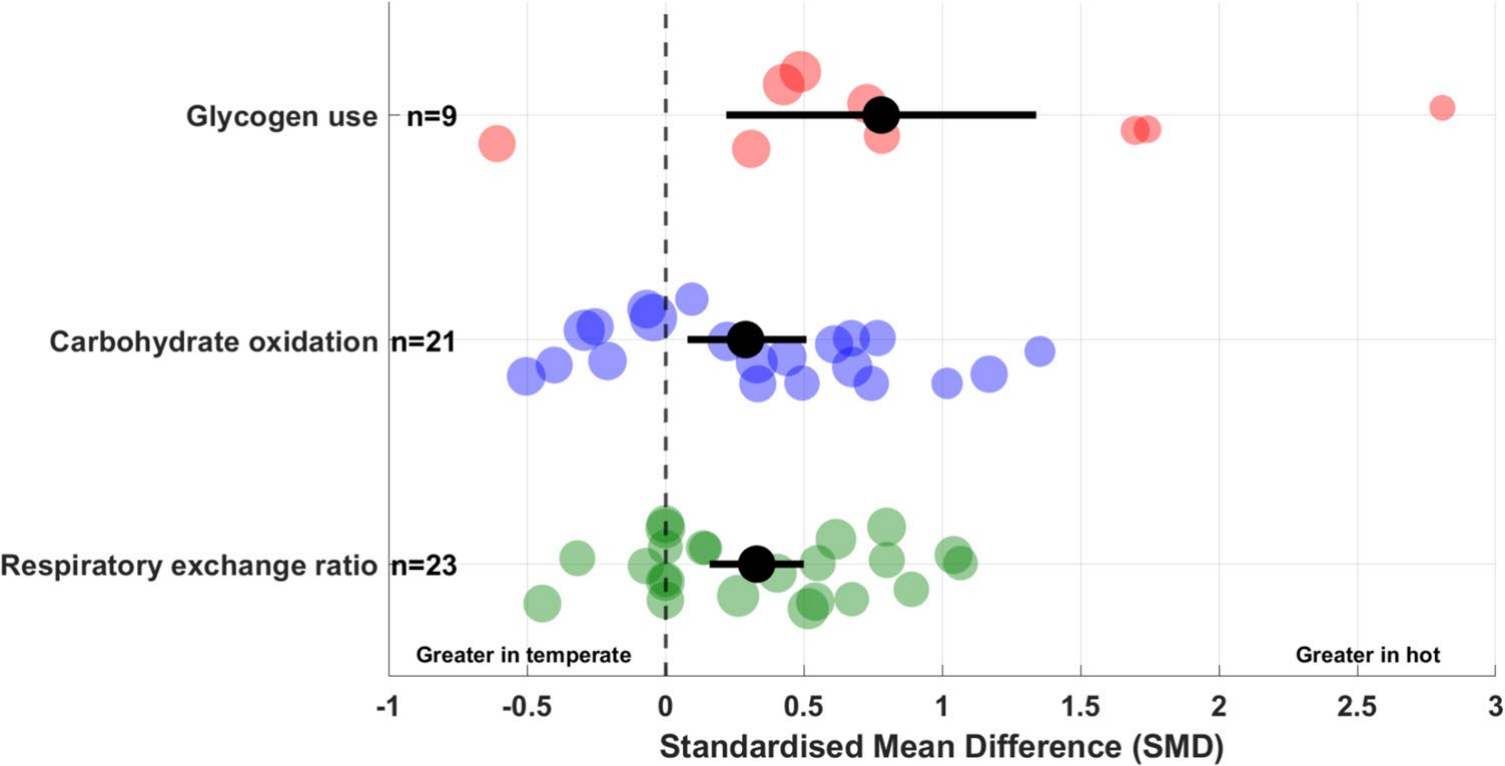
Raw effect size of the effects of prolonged exercise in hot vs. temperate conditions on glycogen use (red), carbohydrate oxidation (blue) and respiratory exchange ratio (green). Pooled standardised mean differences (SMDs) with 95% confidence intervals (horizontal bar) for each variable are represented in black. Each dot represents an individual study and its effect size, with the size reflecting study precision. The number of studies included in each analysis is presented next to the respective data points (left part of the figure)

Figure 5 shows the raw effect sizes for the effects of exercising in hot versus temperate conditions on glycogen use, carbohydrate oxidation and RER.

### The Effect of Prolonged Exercise in Hydrated Versus Dehydrated Status on RER, Carbohydrate Oxidation and Glycogen Use

**Fig. 6:**
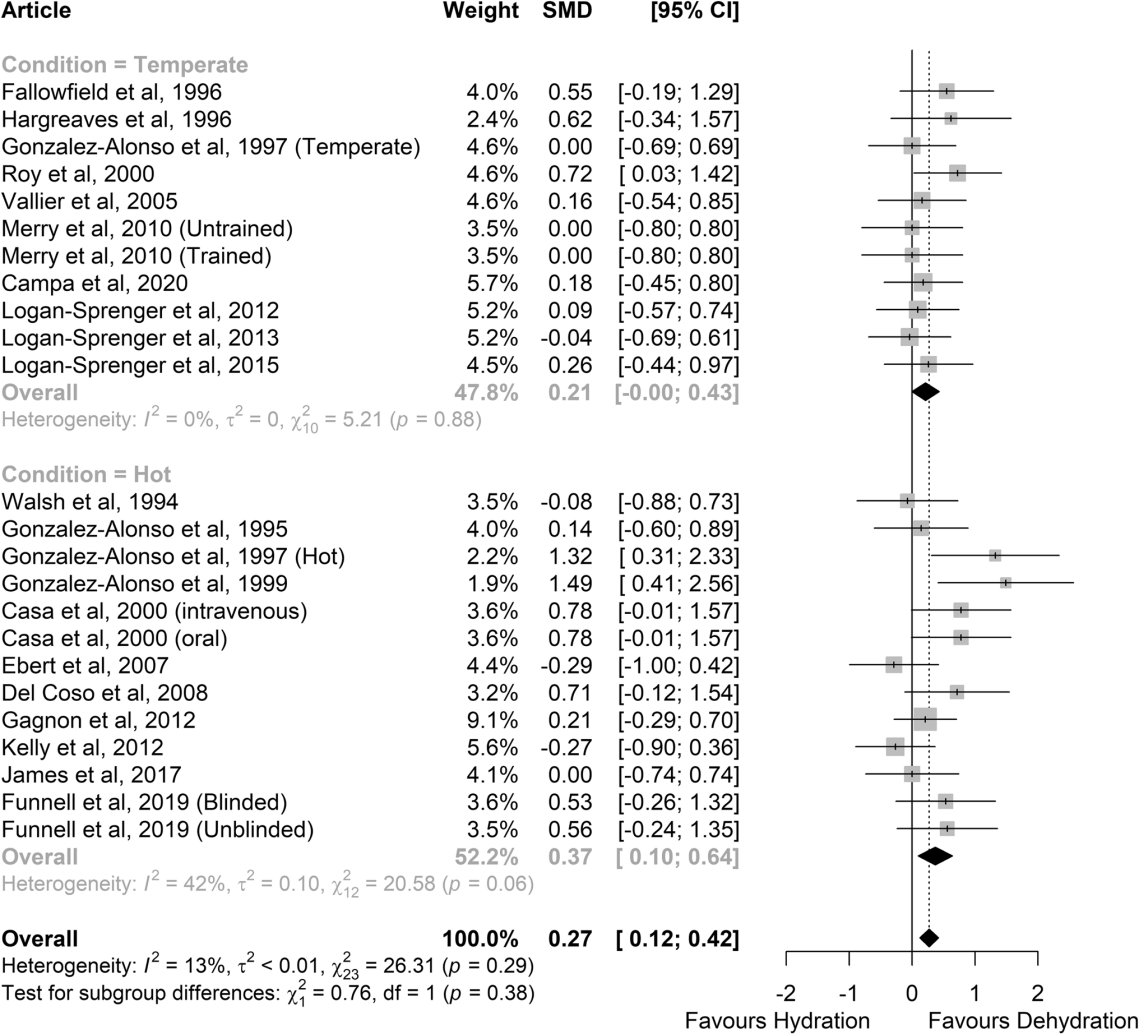
Comparison of the effects of prolonged exercise in hydrated vs. dehydrated status on the respiratory exchange ratio. The lower part shows the studies performed in hot conditions (≥ 28 °C); the upper part shows studies performed in temperate conditions. Forest plot shows standardised mean differences (SMDs) with 95% confidence intervals (CIs). Squares represent the SMD for each study. The diamond represents the pooled SMD for all studies. *df* degrees of freedom

Following data pooling, a greater RER was found in a dehydrated state (SMD 0.27, 95% CI 0.12–0.42; *P* = 0.018; *Z* = 2.37; *small* effect; Fig. 6). However, the effect was only observed in hot (*N* = 13, SMD 0.37, 95% CI 0.10–0.64; *P* = 0.008; *Z* = 2.65; *small* effect) and not in temperate conditions (*N* = 11, SMD 0.21, 95% CI − 0.00 to 0.43; *P* = 0.053; *Z* = 1.94; *small* effect). Heterogeneity was significant in hot conditions (*I*^2^ = 42%; *P* = 0.06) with a moderate risk, but not in temperate conditions (*I*^2^ = 0%; *P* = 0.88).

**Fig. 7:**
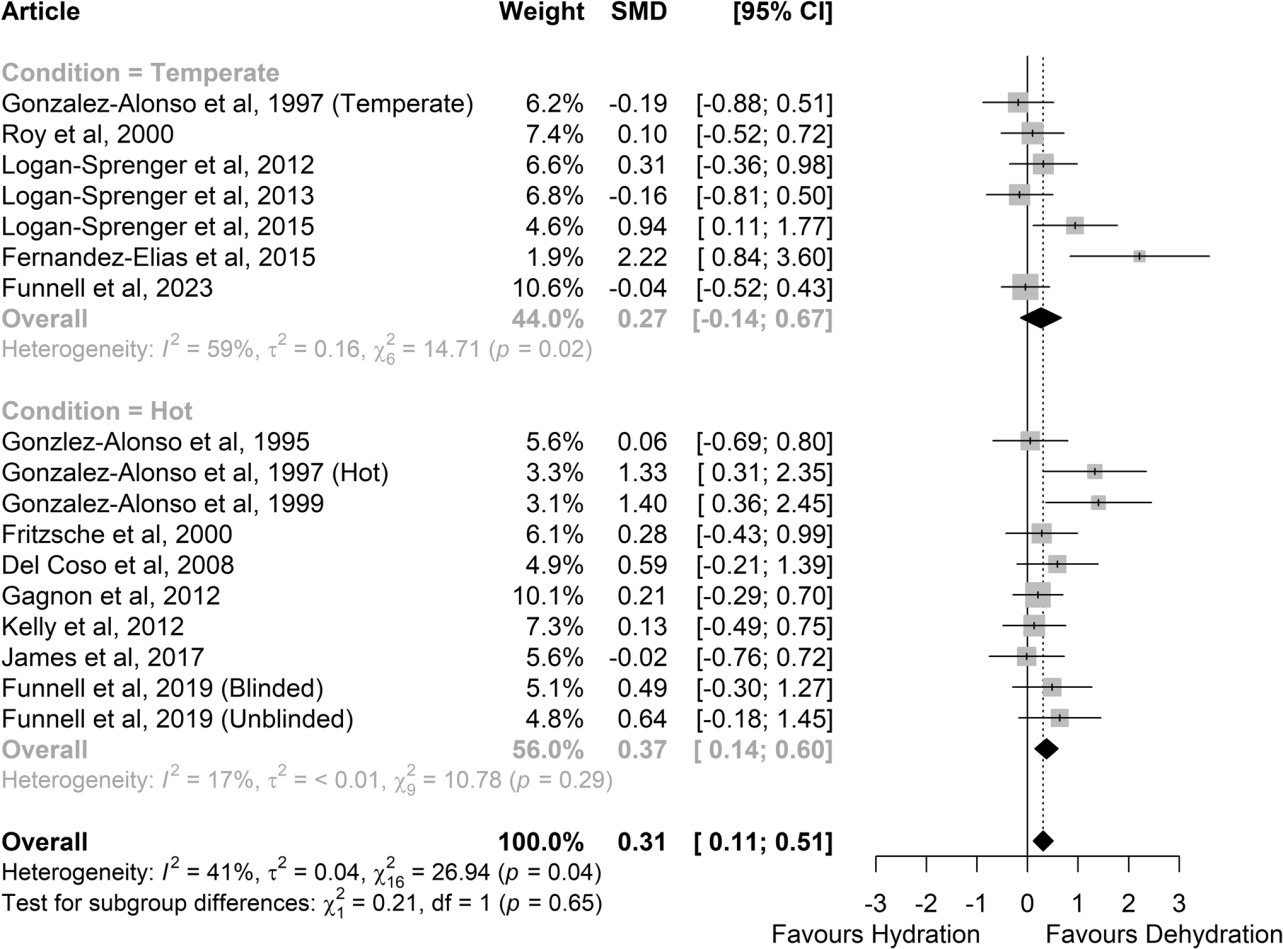
Comparison of the effects of prolonged exercise in hydrated vs. dehydrated status on carbohydrate oxidation. The lower part shows the studies performed in hot conditions (≥ 28 °C); the upper part shows studies performed in temperate conditions. Forest plot shows standardised mean differences (SMDs) with 95% confidence intervals (CIs). Squares represent the SMD for each study. The diamond represents the pooled SMD for all studies. *df* degrees of freedom

Greater carbohydrate oxidation was observed in a dehydrated versus hydrated status (SMD 0.31 (95% CI 0.11–0.51; *small* effect; *P* = 0.002; *Z* = 3.09; Fig. 7). However, the effect was only observed in hot (*N* = 10, SMD 0.37, 95% CI 0.14–0.60; *small* effect; *P* = 0.001; *Z* = 3.18; *small* effect) and not in temperate conditions (*N* = 7, SMD 0.27, 95% CI − 0.14 to 0.67; *P* = 0.199; *Z* = 1.28). However, heterogeneity was significant with a moderate risk in temperate conditions (*I*^2^ = 59%; *P* = 0.02), but not in hot conditions (*I*^2^ = 17%; *P* = 0.29).

**Fig. 8:**
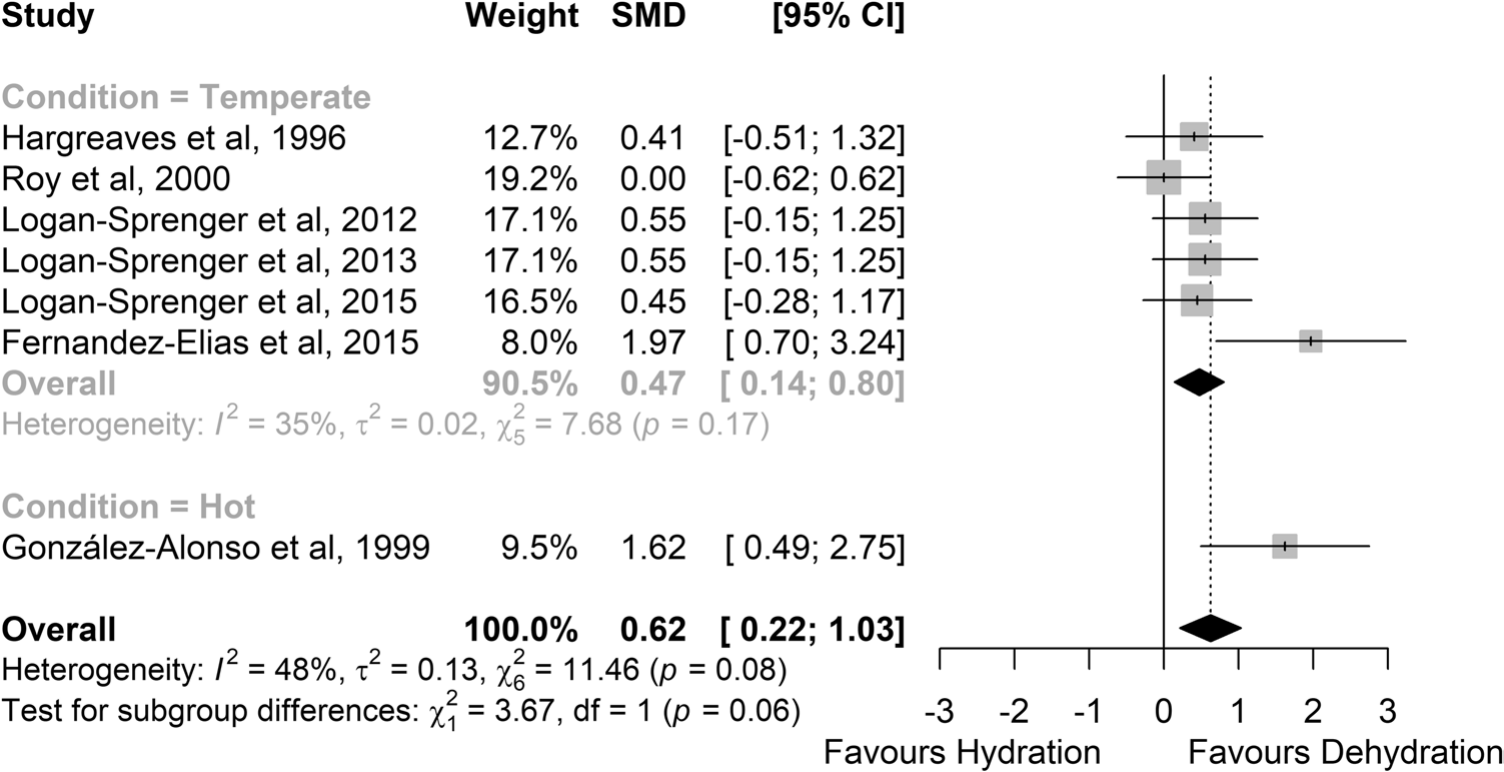
Comparison of the effects of prolonged exercise in hydrated vs. dehydrated status on glycogen use. The lower part shows the studies performed in hot conditions (≥ 28 °C); the upper part shows studies performed in temperate conditions. Forest plot shows standardised mean differences (SMDs) with 95% confidence intervals (CIs). Squares represent the SMD for each study. The diamond represents the pooled SMD for all studies. *df* degrees of freedom

There was greater glycogen use in dehydrated versus hydrated status (SMD 0.62, 95% CI 0.22–1.03; *medium* effect; *P* = 0.003; *Z* = 2.99; Fig. 8). The effect was also observed in temperate conditions (*N* = 6, SMD 0.47, 95% CI 0.14–0.80; *P* = 0.005; *Z* = 2.82; *small* effect). In hot conditions, only one study was conducted, therefore preventing inferential statistics. Heterogeneity was not significant in temperate conditions, but a low risk was detected (*I*^2^ = 35%; *P* = 0.17).

**Fig. 9:**
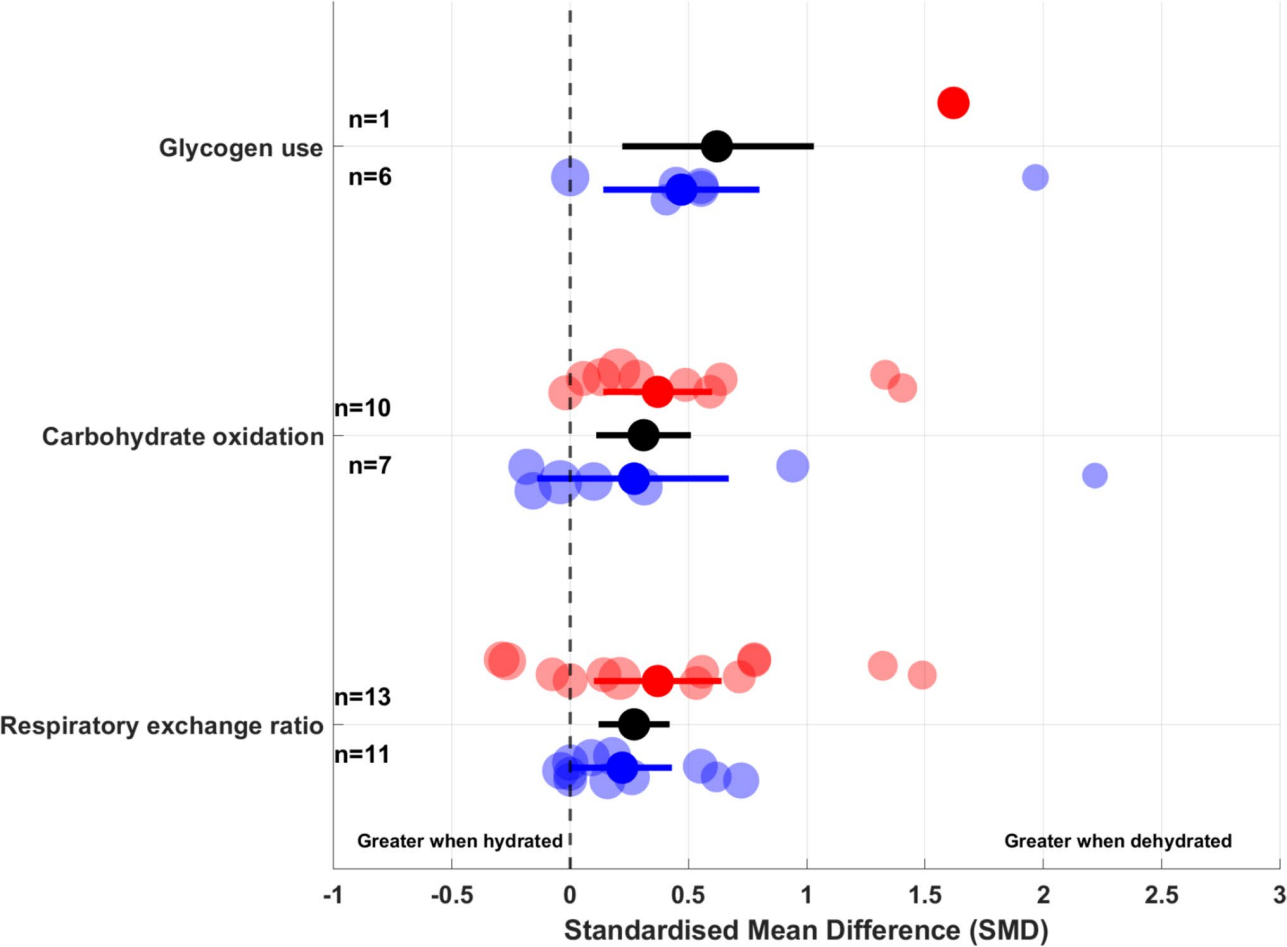
Raw effect size of the effects of prolonged exercise in hydrated vs. dehydrated status on glycogen use, carbohydrate oxidation and respiratory exchange ratio across hot (red) and temperate (blue) conditions. Pooled standardised mean differences (SMDs) with 95% confidence intervals (horizontal bar) for each variable are represented in black. Pooled SMDs with 95% confidence intervals (horizontal bars) for hot conditions are presented in red, and in blue for temperate conditions. Each dot represents an individual study and its effect size, with the size reflecting study precision. The number of studies included in each analysis is presented next to the respective data points (left part of the figure)

Figure 9 shows the raw effect size of the effects of exercising in hydrated versus dehydrated status on glycogen use, carbohydrate oxidation and RER, across hot and temperate conditions.

## Discussion

Carbohydrate oxidation is increased during prolonged exercise in hot compared to temperate conditions (SMD 0.29, *P* = 0.006). The effects of dehydration are more variable, particularly in temperate conditions, where increased carbohydrate oxidation is not consistently observed (temperate: SMD 0.27, *P* = 0.199; hot: SMD 0.37, *P* = 0.001), while increased glycogen use has been observed regardless of the environmental condition when dehydrated (SMD 0.62, *P* = 0.003).

### Impact of Heat Exposure

Irrespective of the indices observed (i.e. carbohydrate oxidation or RER), the results of these meta-analyses demonstrate that heat exposure during prolonged endurance exercise increases carbohydrate utilisation. Interestingly, as well as showing greater carbohydrate oxidation at the whole-body level, the effects of heat stress have also been demonstrated on muscle glycogen use. In 1975, Fink et al. [7] were the first to report increased muscle glycogen use in hot conditions, before these findings were widely confirmed during constant-intensity exercises [5, 6, 18, 50]. These results support a shift towards greater carbohydrate use and decreased lipid use compared to thermoneutral conditions [13], and are supported by an increased hepatic glucose output [10] and higher lactate accumulation [5–8, 10, 18, 44, 50]. However, effects of heat exposure seem to be greater at moderate intensity [24, 56], since no difference in muscle glycogen breakdown between hot and cool environments was observed during supra-maximal exercise [67], despite higher lactate concentrations [68].

Some studies have failed to demonstrate an effect of hot conditions on carbohydrate utilisation, as reflected by a significant heterogeneity in the meta-analysis examining carbohydrate oxidation in hot versus temperate conditions. Several methodological factors could explain these discrepancies, including the exercise intensity used, the difference in ambient temperature between trials and/or the lack of difference between conditions for body core temperatures. For example, Cheuvront and Haymes [33] did not report any difference in rectal temperature or carbohydrate oxidation at the end of prolonged exercise. Galloway and Maughan [48] did not report any difference in carbohydrate oxidation between conditions despite significant differences in rectal temperature; however, in both conditions, rectal temperature exceeded 39 ºC, even in temperate conditions. Consequently, hyperthermia may have affected both conditions, making the carbohydrate oxidation similar regardless of the differences in ambient temperature. Regarding glycogen use, only one study failed to observe greater glycogen use in hot conditions following prolonged exercise [39], but this may be attributed to lower pre-exercise muscle glycogen concentrations in the hot condition (i.e. 611 vs. 470 mmol glucose/kg dry tissue at rest before exercise for temperate and hot conditions, respectively). These differences in muscle glycogen concentrations pre-exercise could be explained by the lack of dietary control prior to the experiments. The results could suggest that the increased carbohydrate use observed in hot conditions may be primarily driven by elevated body temperature, although this has not been directly analysed in the present study. This is supported by evidence showing that interventions aiming at reducing body core and muscle temperature in hot conditions, such as heat acclimation [6, 69, 70] and cooling [71], appear to mitigate the effects of heat stress on substrate utilisation, with reduced carbohydrate use.

Several mechanisms are likely to explain the increased use of endogenous carbohydrate during endurance exercise under heat stress [23]. One of the primary responses to elevated body core temperature is increased skin blood flow, promoting heat loss through evaporation [14, 23]. This leads to a dual demand on the cardiovascular system: (1) meeting metabolic requirements for oxygen delivery in active skeletal muscles and (2) facilitating thermoregulation and evaporative loss by increasing the skin blood flow [72]. This dual demand reduces blood flow to active muscles, limiting oxygen delivery during exercise in the heat [73, 74], which may decrease the use of aerobic pathways and lipid use. Although the reduction in oxygen supply to active muscles in hot conditions has been reported for several years [73, 74], effects on oxygen use remain unclear as arterio-venous oxygen difference is increased in hot conditions [3], showing enhanced oxygen extraction. Although oxygen supply is unlikely to be the main factor influencing substrate utilisation during exercise in the heat, the influence of decreased muscle blood flow on several metabolic mechanisms cannot be excluded. For example, an altered muscle blood flow could reduce nutrient supply and removal of metabolic by-products [75].

One other mechanism that may impact carbohydrate oxidation is the increase in circulating epinephrine concentrations during exercise [76], and this rise is further amplified when exercise is conducted in hot environments [5, 9, 73]. As glycogen phosphorylase is sensitive to β-adrenergic receptor stimulation [77], increased circulating epinephrine is likely to increase intramuscular glycogen utilisation. Consequently, the simultaneous increase in carbohydrate utilisation and circulating epinephrine concentrations may represent an additional factor explaining the greater reliance on carbohydrate utilisation during prolonged exercise in the heat, as observed in previous studies [5, 17, 56, 78]. This hypothesis is supported by studies demonstrating increased muscle glycogen use in individuals infused with epinephrine while exercising [79]. Moreover, elevated epinephrine concentrations could also play an important role on circulating glucose and hepatic glucose production [80, 81]; potentially explaining the hyperglycaemia reported during prolonged exercise in hot conditions [5, 7, 68, 82].

Finally, heat exposure typically results in increased dehydration due to evaporative losses aimed at reducing body core temperature. This dehydration reduces cellular water content and cell volume, potentially disrupting cell metabolism and triggering catabolic responses, ultimately increasing carbohydrate utilisation [83–85]. These dehydration effects, exacerbated by heat exposure (and not always well controlled, as only 16 of the 29 studies comparing hot and temperate conditions reported body mass changes), are discussed below.

### Impact of Dehydration

In addition to the effects of environmental conditions, dehydration, whether induced by heat exposure or not (i.e. dehydration can occur in the absence of environmental stressors), may also influence substrate utilisation. Recent studies show that dehydration, with associated body water losses, increased glycogen use during prolonged exercise [18–21]. Moreover, previous animal studies have shown that a reduction in muscle water content may stimulate glycogenolysis during exercise. In rat liver, for example, tissue dehydration favoured a range of catabolic responses, including glycogenolysis [83–85]. These effects observed in animals have not been confirmed in humans yet.

The findings of the present study partially support this hypothesis with significant effects observed on all indices (i.e. RER, carbohydrate oxidation and glycogen use). However, significant heterogeneity was observed for RER and carbohydrate oxidation, indicating discrepancies in the results. Interestingly, a subgroup analysis on environmental conditions revealed distinct differences between studies conducted in temperate and hot conditions. Carbohydrate oxidation was increased under dehydration in hot (SMD 0.37, *P* = 0.001) but not temperate conditions (SMD 0.27, *P* = 0.199). These results, observed for RER and carbohydrate oxidation, suggest that dehydration, in the absence of hyperthermia induced by hot conditions, does not significantly affect carbohydrate utilisation, even though most studies show reduced plasma volume, which is likely to impair blood volume and muscle blood flow. Greater muscle glycogen use was observed even under temperate conditions. However, the lower methodological quality of six out of seven of these studies (i.e. studies that did not meet 60% quality based on risk of bias [Supplementary material 1; Table 2] or were non-randomised) raises concerns about the reliability of these findings.

In hot conditions, the impact of dehydration seems more pronounced, through several potential mechanisms. Firstly, maintaining hydration in hot conditions may help prevent the body core temperature rising above a certain level, particularly if the drink ingested is cooler than body temperature, limiting some physiological responses linked to elevated muscle temperature (i.e. enzyme activities, circulating epinephrine). Secondly, hot conditions favour a blood flow redistribution that compromises oxygen and glucose delivery to muscles. In hot conditions, a reduced blood volume caused by dehydration exacerbates the challenge posed by the dual demand for blood flow between active muscles and the skin for evaporative losses. This mechanism could then create an energy imbalance (due to reduced oxygen availability) that stimulates glycogen phosphorylase [18]. However, Gonzalez-Alonso et al. [9] reported that despite a reduced muscle blood flow with dehydration, no difference was observed on leg oxygen consumption and glucose delivery between hydrated and dehydrated statuses, questioning the impact of this mechanism on substrate use. Lastly, in most studies, the levels of dehydration observed (as indicated by the reduction in body mass) were greater in hot conditions compared to temperate conditions. This disparity may lead to a greater reduction in cellular water content and, consequently, enhanced glycogenolysis, as described in animal studies [83–85].

Fernández-Elias et al. [18] demonstrated that the increased glycogen use with dehydration during prolonged exercise in temperate conditions was accompanied by an increase in body core temperature, significantly higher than in euhydrated conditions. In a subsequent trial, the authors manipulated ambient temperature in euhydrated conditions to achieve a similar rise in body core temperature as observed during exercise under dehydrated conditions. Interestingly, when the rise in body core temperature was comparable despite differences in hydration status, muscle glycogen use did not differ between conditions. However, variations in ambient temperature alter external stimuli, possibly influencing thermoregulatory responses, which requires caution in interpreting these results. These findings support that dehydration, in the absence of differences in body core temperature, does not influence carbohydrate use during prolonged exercise. Interestingly, these findings align with evidence of greater carbohydrate oxidation in hot compared to temperate conditions in studies where hydration levels were similar between conditions. Fernández-Elias et al. [18] suggested that a magnitude of muscle dehydration of 4.6 ± 0.3% was not a stronger stimulus than hyperthermia (i.e. 39.2 ± 0.15 °C, gastrointestinal temperature) to increase intramuscular carbohydrate utilisation [18].

The discrepancies/heterogeneity in the effects of hydration status on substrate use in the heat (e.g. for RER) may be explained by the differences in hydration strategies used. Factors such as the method of fluid administration (e.g. oral ingestion or intravenous infusion) and the temperature of the fluid ingested may promote or prevent a reduction in body core temperature via a cooling effect [40]. For example, Casa et al. [40] reported that oral ingestion of cool water led to a lower body core temperature compared to intravenous fluid infusion, potentially due to the cooler temperature of the ingested compared to the infused water.

### Limitations and Perspectives

In hot conditions, athletes often adjust their workload to prevent excessive rises in body core temperature rather than maintaining it until exhaustion [33, 86]. Consequently, the use of self-selected/adapted workloads in studies is essential for ensuring ecological validity and translational applicability; however, this aspect remains understudied in experimental designs. Further studies should investigate this type of exercise. Recent studies have used heart rate to standardise exercise intensity between different environmental conditions (matched heart rate), showing a lower external workload in heat, which subsequently leads to reduced carbohydrate utilisation [78]. While matching heart rate is interesting from an ecological perspective, it remains a contentious technique. Changes in heart rate during hot conditions do not necessarily reflect alterations in oxygen utilisation or relative exercise intensity, as these variations may primarily arise from the redistribution of blood flow. Consequently, further studies should prioritise matching oxygen consumption for more accurate comparisons. Such research could provide valuable insights into exercise performance, particularly in contexts where glycogen depletion is a critical determinant. By mitigating the effects of hyperthermia on fatigue [9] due to a workload reduction, these studies could improve our understanding of the role of carbohydrate in performance in hot conditions.

Although endogenous carbohydrate use has been shown to increase in the heat, previous studies have reported a decrease in exogenous carbohydrate oxidation [50], adding complexity to the understanding of carbohydrate use in hot conditions. This reduction may be attributed to factors such as decreased gastric emptying and/or intestinal absorption due to reduced splanchnic blood flow [50, 72, 87, 88]. Furthermore, although the impact of water deficit on endogenous carbohydrate use remains debated, dehydration may significantly affect the use of exogenous carbohydrates by slowing gastric emptying [89]. Further studies should explore the use of exogenous carbohydrate in a dehydrated condition, in both temperate and hot conditions.

Meta-regressions examining the effects of core temperature and body mass loss have been performed (Supplementary material 4), but differences in methodology (e.g. the various techniques used to measure core temperature) strongly limit the strength and interpretation of these findings.

Finally, several mechanisms possibly explaining the increased carbohydrate use in hot conditions are related to responses at the muscular level. Therefore, it would be prudent to consider muscle temperature in addition to core (rectal or gastrointestinal) and skin temperatures in future studies.

## Conclusion

Carbohydrate utilisation increases during prolonged exercise in hot conditions. However, dehydration (up to ~ 4% body mass loss) does not appear to significantly impact carbohydrate use, provided that body core temperature remains unaffected. The primary factor driving the rise in carbohydrate utilisation appears to be the elevated body core and likely elevated muscle temperature, specifically. These findings indicate that rehydration may not be the most important strategy to limit carbohydrate use during exercise, particularly in temperate conditions; rather, implementing cooling strategies to manage body core temperature could be as effective, especially in activities where the possibility of fluid intake can be limited (e.g. running).

## Supplementary Information

Below is the link to the electronic supplementary material.

Supplementary file1 (PDF 254 KB)

Supplementary file2 (PDF 979 KB)

Supplementary file3 (PDF 210 KB)

Supplementary file4 (PDF 280 KB)

## Declarations

### Data availability

Data are available on request from the corresponding author.
