# Supplementary material for: The Effect of Heat Stress and Dehydration on Carbohydrate Use During Endurance Exercise: A Systematic Review and Meta-Analysis
Source: Sports Med. 2025 Aug 20;55(11):2825–47. doi: 10.1007/s40279-025-02294-3 (PMC12559103; doi:10.1007/s40279-025-02294-3)
Supplement: Supplementary file 1 — Supplementary file1 (PDF 254 KB) [file 40279_2025_2294_MOESM1_ESM.pdf]

Supplementary material 1: Risks of bias

Title: The effect of heat stress and dehydration on carbohydrate use during endurance exercise: A systematic review and meta-analysis

Journal: Sports Medicine

Running heading: Heat stress and dehydration's impact on carbohydrate use in endurance exercise: A systematic review

Authors: Loïs Mougin[1], Heather Z Macrae[1], Lee Taylor[1], Lewis J James[1], Stephen A Mears[1*].

Affiliation(s):

[1] National Centre for Sport and Exercise Medicine, School of Sport, Exercise and Health Sciences, Loughborough University, Loughborough, United Kingdom.

***Corresponding author**

Stephen A Mears, School of Sport, Exercise and Health Sciences, National Centre for Sport and Exercise Medicine, Loughborough University, Loughborough, Leicestershire LE11 3TU, UK

Email: s.a.mears@lboro.ac.uk ; Phone: (+44) 1509 226391

Table 1. Risk of bias scores for the heat part assessed using van Rosendale Scale for the studies included in this systematic review. Percentage score was calculated by dividing the number of yes scores (+) by the total number of applicable items. + = yes; − = no/ not sure; N/A = not applicable.

	Fink et al, 1975	Young et al, 1985	Dolny et al, 1988	Barnett et al, 1993	Snow et al, 1993	Nielsen et al, 1994a	Nielsen et al, 1994b	Febbraio et al, 1994a	Febbraio et al, 1994b	Young et al, 1995	Hargreaves et al, 1996	Galloway et al, 1997	Cheuvront et al, 2001	Marino et al, 2001	Nybo et al, 2001	Jentjens et al, 2002	Yamashita et al, 2005	Hayashi et al, 2006	Hettinga et al, 2007	Shorten et al, 2009	Fernandez-Elias et al, 2015	O'Hearn et al, 2016	Collins et al, 2017	Hoffman et al, 2018	Maunder et al, 2020	Foster et al, 2023	Scheerberlein et al, 2023	Mora-rodriguez et al, 2024	Rosbrook et al, 2024
A clear description of the inclusion and exclusion criteria was provided	+	-	-	+	+	+	+	+	-	+	-	-	+	+	+	+	-	-	+	+	+	+	+	-	+	+	+	+	+
The trials were randomised	-	-	+	-	+	+	+	+	+	-	-	+	+	+	+	+	+	+	-	+	+	+	+	+	+	+	+	+	+
The method used to generate the random allocation sequence, including details of any restrictions (e.g. blocking, stratification) was described	N/A	N/A	-	N/A	-	-	-	+	-	N/A	N/A	+	-	+	+	-	-	-	N/A	+	+	+	+	-	+	-	+	+	+
Sample size was justified (e.g. by power calculation)	-	-	-	-	-	-	-	-	-	-	-	-	+	-	-	-	-	-	-	-	-	-	-	+	-	+	-	+	-
Attempts were made to control and/or monitor pre-trial conditions (e.g. diet, exercise)	-	+	+	+	+	+	+	+	+	+	+	+	+	+	+	+	-	+	+	+	+	+	+	-	+	+	+	+	+
Design incorporated measures of important baseline variables	+	+	+	+	+	+	+	+	+	+	+	+	+	+	+	+	+	+	+	+	+	+	+	+	+	+	+	+	+
There was blinding of all subjects	N/A	N/A	N/A	N/A	N/A	N/A	N/A	N/A	N/A	N/A	N/A	N/A	N/A	N/A	N/A	N/A	N/A	N/A	N/A	N/A	N/A	N/A	N/A	N/A	N/A	N/A	N/A	N/A	N/A
There was blinding of all investigators involved in the trials	N/A	N/A	N/A	N/A	N/A	N/A	N/A	N/A	N/A	N/A	N/A	N/A	N/A	N/A	N/A	N/A	N/A	N/A	N/A	N/A	N/A	N/A	N/A	N/A	N/A	N/A	N/A	N/A	N/A
Both the method of blinding and the evaluation of the successfulness of blinding were described	N/A	N/A	N/A	N/A	N/A	N/A	N/A	N/A	N/A	N/A	N/A	N/A	N/A	N/A	N/A	N/A	N/A	N/A	N/A	N/A	N/A	N/A	N/A	N/A	N/A	N/A	N/A	N/A	N/A
Details were provided regarding the inability of a subject to complete study requirements	+	N/A	N/A	N/A	N/A	N/A	N/A	N/A	+	N/A	N/A	N/A	N/A	N/A	+	N/A	N/A	+	N/A	N/A	N/A	N/A	N/A	N/A	N/A	N/A	N/A	N/A	N/A
Statistical methods used to compare groups for primary outcome measure(s),b and methods for additional analyses, such as subgroup analyses and adjusted analyses, were described	+	+	+	+	+	+	+	+	+	+	+	+	+	+	+	+	+	+	+	+	+	+	+	+	+	+	+	+	+
Both point measures and measures of variability for the primary outcome measure(s)a were provided	+	+	+	+	+	+	+	+	+	+	+	+	+	+	+	+	+	+	+	+	+	+	+	+	+	+	+	+	+
The results of between-group statistical comparisons were reported for the primary outcome measure(s)b [e.g. an estimated effect size], and its precision (e.g. 95% CI)	+	+	+	+	+	+	+	+	+	+	+	+	+	+	+	+	+	+	+	+	+	+	+	+	+	+	+	+	+
The method used to assess adverse effects was described	+	+	+	+	+	+	+	+	+	+	+	+	+	+	+	+	+	+	+	+	+	+	+	+	+	+	+	+	+
Reproducibility of the primary outcome measure(s)b was reported	-	-	-	-	-	-	-	-	-	-	-	-	-	-	-	-	-	-	-	-	+	-	-	-	-	-	-	-	-
If a performance test was used, a familiarisation trial was conducted	N/A	N/A	N/A	N/A	N/A	N/A	N/A	N/A	N/A	N/A	N/A	+	N/A	+	N/A	N/A	N/A	+	N/A	N/A	N/A	N/A	N/A	N/A	N/A	N/A	N/A	N/A	N/A
Total score (%)	64	60	64	70	73	73	73	82	67	70	60	75	82	83	83	73	55	69	70	82	91	52	52	64	82	82	82	91	82

Table 2. Risk of bias scores for the dehydration part assessed using van Rosendale Scale for the studies included in this systematic review. Percentage score was calculated by dividing the number of yes scores (+) by the total number of applicable items. + = yes; − = no/ not sure; N/A = not applicable.

	Walsh et al, 1994	González-Alonso et al, 1995	Fallowfield et al, 1996	Hargreaves et al, 1996	González-Alonso et al, 1997	González-Alonso et al, 1999	Casa et al, 2000	Fritzsche et al, 2000	Roy et al, 2000	Vallier et al, 2005	Ebert et al, 2007	Del Coso et al, 2008	Merry et al, 2010	Gagnon et al, 2012	Kelly et al, 2012	Logan-Sprenger et al, 2012	Logan-Sprenger et al, 2013	Fernandez-Elias et al, 2015	Logan-Sprenger et al, 2015	James et al, 2017	Funnell et al, 2019	Campa et al, 2020	Funnell et al, 2023
A clear description of the inclusion and exclusion criteria was provided	-	+	+	-	+	+	+	-	-	+	-	-	+	+	+	-	-	+	-	+	+	+	+
The trials were randomised	+	+	+	-	+	-	+	+	+	-	+	+	+	+	+	+	+	+	+	+	+	+	+
The method used to generate the random allocation sequence, including details of any restrictions (e.g. blocking, stratification) was described	+	-	-	N/A	+	N/A	+	+	-	N/A	+	-	+	-	+	-	-	+	-	-	+	+	-
Sample size was justified (e.g. by power calculation)	-	-	-	-	-	-	-	-	-	-	-	-	-	-	-	-	-	-	-	+	+	-	+
Attempts were made to control and/or monitor pre-trial conditions (e.g. diet, exercise)	+	+	+	+	+	+	+	+	+	+	+	+	+	+	+	+	+	+	+	+	+	+	+
Design incorporated measures of important baseline variables	+	+	+	+	+	+	+	+	+	+	+	+	+	+	+	+	+	+	+	+	+	+	+
There was blinding of all subjects	-	-	-	-	-	-	-	-	-	-	-	-	-	-	-	-	-	-	-	+	+	-	-
There was blinding of all investigators involved in the trials	N/A	N/A	N/A	N/A	N/A	N/A	N/A	N/A	N/A	N/A	N/A	N/A	N/A	N/A	N/A	N/A	N/A	N/A	N/A	N/A	N/A	N/A	N/A
Both the method of blinding and the evaluation of the successfulness of blinding were described	N/A	N/A	N/A	N/A	N/A	N/A	N/A	N/A	N/A	N/A	N/A	N/A	N/A	N/A	N/A	N/A	N/A	N/A	N/A	+	-	N/A	N/A
Details were provided regarding the inability of a subject to complete study requirements	N/A	N/A	N/A	N/A	N/A	N/A	N/A	N/A	N/A	N/A	N/A	N/A	N/A	N/A	N/A	N/A	N/A	N/A	N/A	N/A	N/A	N/A	N/A
Statistical methods used to compare groups for primary outcome measure(s),b and methods for additional analyses, such as subgroup analyses and adjusted analyses, were described	+	+	+	+	+	+	+	+	+	+	+	+	+	+	+	+	+	+	+	+	+	+	+
Both point measures and measures of variability for the primary outcome measure(s)a were provided	+	+	+	+	+	+	+	+	+	+	+	+	+	+	+	+	+	+	+	+	+	+	+
The results of between-group statistical comparisons were reported for the primary outcome measure(s)b [e.g. an estimated effect size], and its precision (e.g. 95% CI)	+	+	+	+	+	+	+	+	+	+	+	+	+	+	+	+	+	+	+	+	+	+	+
The method used to assess adverse effects was described	+	+	+	+	+	+	+	+	+	+	+	+	+	+	+	+	+	+	+	+	+	+	+
Reproducibility of the primary outcome measure(s)b was reported	-	-	-	-	-	-	-	-	-	-	-	-	-	-	-	-	-	-	-	-	-	-	-
If a performance test was used, a familiarisation trial was conducted	-	N/A	+	N/A	N/A	N/A	N/A	+	N/A	N/A	+	+	+	N/A	N/A	N/A	N/A	+	N/A	+	+	-	+
Total score (%)	62	67	69	50	75	64	75	69	58	58	69	67	77	67	75	58	58	77	58	86	86	69	77
