# Supplementary material for: The Effect of Heat Stress and Dehydration on Carbohydrate Use During Endurance Exercise: A Systematic Review and Meta-Analysis
Source: Sports Med. 2025 Aug 20;55(11):2825–47. doi: 10.1007/s40279-025-02294-3 (PMC12559103; doi:10.1007/s40279-025-02294-3)
Supplement: Supplementary file 2 — Supplementary file2 (PDF 979 KB) [file 40279_2025_2294_MOESM2_ESM.pdf]

Supplementary material 2: Sensitivity analysis

Title: The effect of heat stress and dehydration on carbohydrate use during endurance exercise: A systematic review and meta-analysis

Journal: Sports Medicine

Running heading: Heat stress and dehydration's impact on carbohydrate use in endurance exercise: A systematic review

Authors: Loïs Mougin[1], Heather Z Macrae[1], Lee Taylor[1], Lewis J James[1], Stephen A Mears[1*].

Affiliation(s):

[1] National Centre for Sport and Exercise Medicine, School of Sport, Exercise and Health Sciences, Loughborough University, Loughborough, United Kingdom.

***Corresponding author**

Stephen A Mears, School of Sport, Exercise and Health Sciences, National Centre for Sport and Exercise Medicine, Loughborough University, Loughborough, Leicestershire LE11 3TU, UK

Email: s.a.mears@lboro.ac.uk ; Phone: (+44) 1509 226391

These analyses exclude studies with low methodological quality (i.e., studies that did not meet 60% quality based on risk of bias (Supplementary material 1; Tables 1 and 2) or were non-randomised.

1. *The effect of prolonged exercise in hot vs. temperate conditions on RER, carbohydrate oxidation and glycogen use*

Greater RER values were found in hot conditions *vs.* temperate conditions (SMD: 0.35, 95% CI 0.14 to 0.56, $P = 0.001$; $Z = 3.22$, *small* effect, Figure 1). In hot conditions, carbohydrate oxidation (SMD: 0.33, 95% CI 0.08 to 0.55, $P = 0.009$; $Z = 2.59$, *small* effect, Figure 2) and glycogen use (SMD = 1.10, 95% CI 0.43 to 1.76; *large* effect; $P = 0.001$; $Z = 3.21$; Figure 3) were higher.

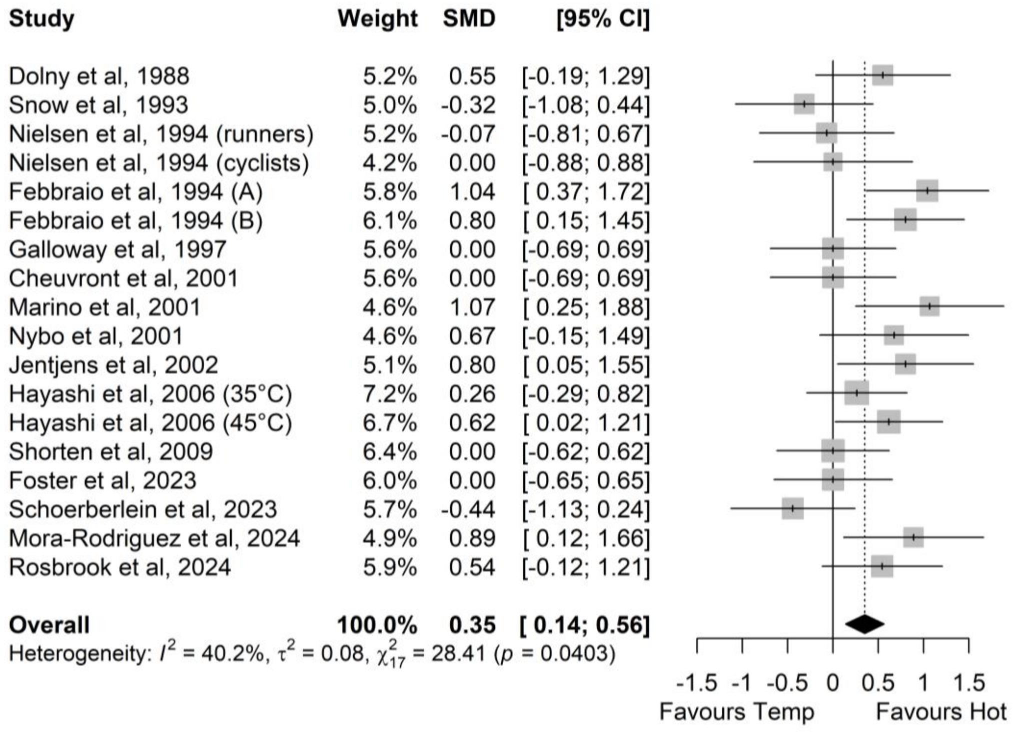

Figure 1. Comparison of the effects of prolonged exercise in Hot *vs.* Temperate (Temp) conditions on the Respiratory Exchange Ratio (RER). Forest plot shows standardised mean differences with 95% confidence intervals. Squares represent the standardised mean difference for each study. The diamond represents the pooled standardised mean difference for all studies. SMD standardised mean difference, CI confidence interval.

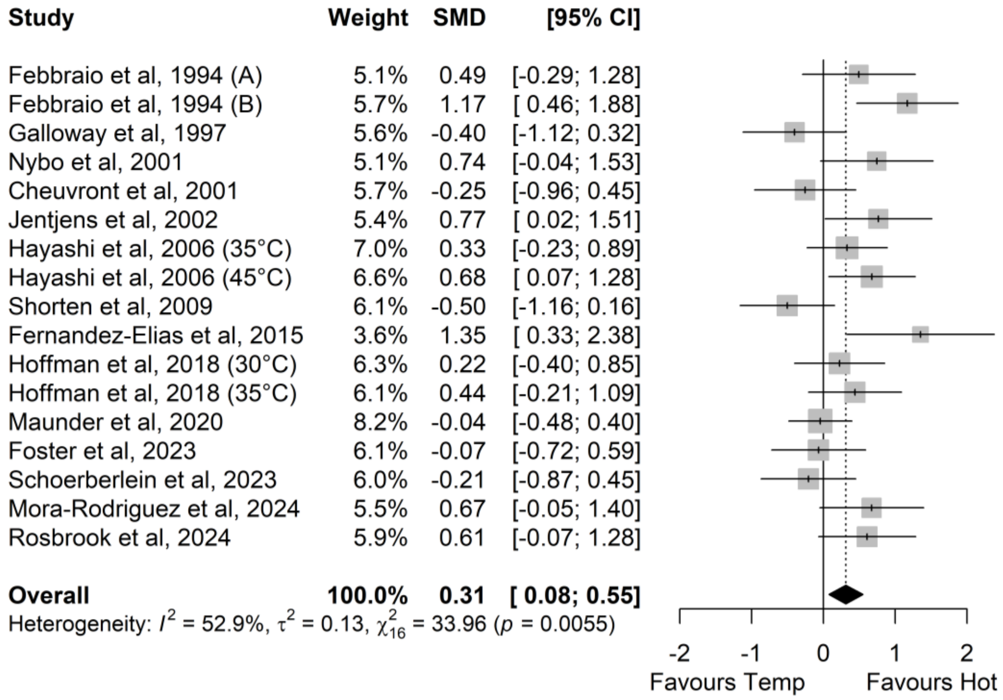

Figure 2. Comparison of the effects of prolonged exercise in Hot *vs*. Temperate (Temp) conditions on carbohydrate oxidation. Forest plot shows standardised mean differences with 95% confidence intervals. Squares represent the standardised mean difference for each study. The diamond represents the pooled standardised mean difference for all studies. SMD standardised mean difference, CI confidence interval.

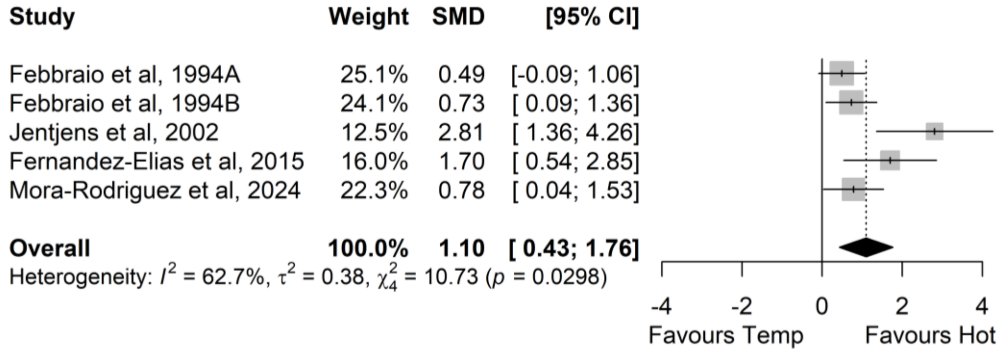

Figure 3. Comparison of the effects of prolonged exercise in Hot *vs.* Temperate (Temp) conditions on glycogen use. Forest plot shows standardised mean differences with 95% confidence intervals. Squares represent the standardised mean difference for each study. The diamond represents the pooled standardised mean difference for all studies. SMD standardised mean difference, CI confidence interval.

2. *The effect of prolonged exercise in hydrated vs. dehydrated status on RER, carbohydrate oxidation and glycogen use.*

A greater RER was found in a dehydrated state (SMD 0.24, 95% CI 0.06 to 0.42; *P* = 0.008; Z = 2.67; *small* effect; Figure 4). However, the effect was only observed in hot (SMD: 0.30, 95% CI 0.05 to 0.55; *P* = 0.008; Z = 2.34; *small* effect) and not in temperate conditions (SMD: 0.15, 95% CI -0.15 to 0.47; *P* = 0.359; Z = 0.92; *trivial* effect).

Greater carbohydrate oxidation was observed in a dehydrated *vs.* hydrated status (SMD: 0.26 (95% CI 0.06 to 0.56; *small* effect; *P* = 0.013; Z = 2.49; Figure 5). However, the effect was only observed in hot (SMD 0.32; 95% CI 0.09 to 0.56; *small* effect; *P* = 0.008; Z = 2.67; *small* effect) and not in temperate conditions (SMD 0.54; 95% CI -0.85 to 1.92; *P* = 0.446; Z = 0.76).

Only one study reported muscle glycogen use in dehydrated condition, consequently, no meta-analysis was performed.

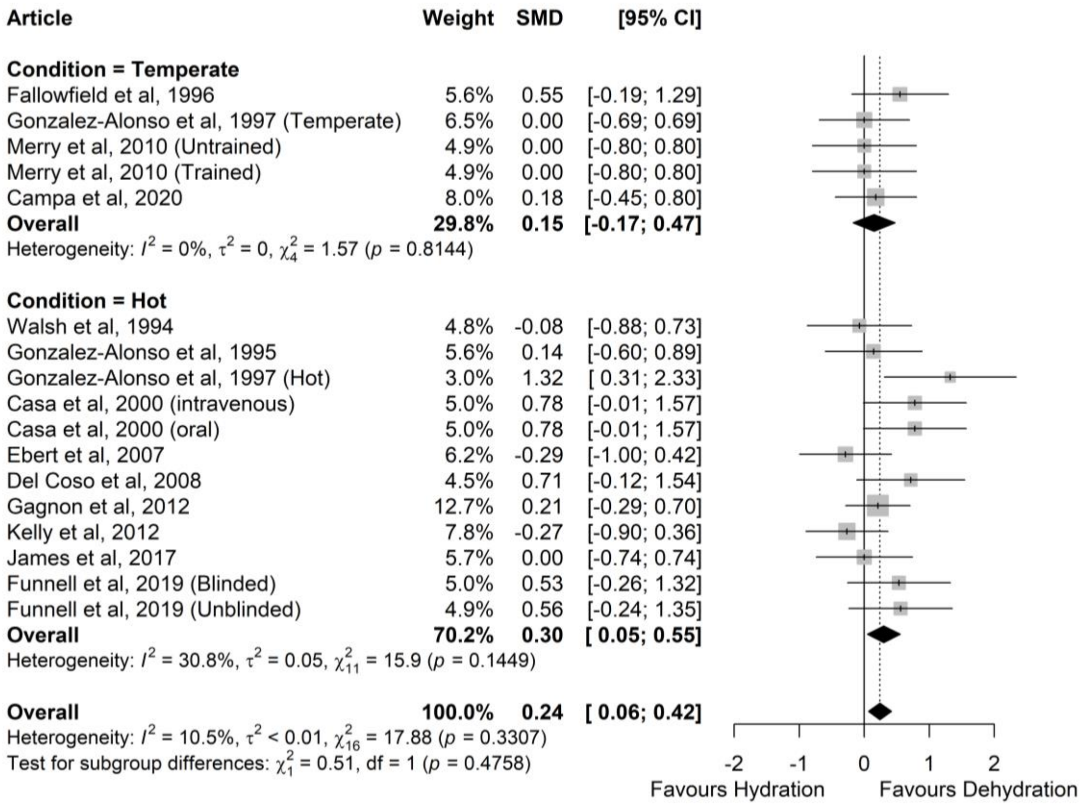

Figure 4. Comparison of the effects of prolonged exercise in hydrated *vs.* dehydrated status on the Respiratory Exchange Ratio. The upper part shows the studies performed in hot conditions (≥ 28°C); The lower part shows studies performed in temperate conditions. Forest plot shows standardised mean differences with 95% confidence intervals. Squares represent the standardised mean difference for each study. The diamond represents the pooled standardised mean difference for all studies. SMD standardised mean difference, CI confidence interval, df degrees of freedom.

Article	Weight	SMD	[95% CI]
**Condition = Temperate**			
Fallowfield et al, 1996	5.6%	0.55	[-0.19; 1.29]
Gonzalez-Alonso et al, 1997 (Temperate)	6.5%	0.00	[-0.69; 0.69]
Merry et al, 2010 (Untrained)	4.9%	0.00	[-0.80; 0.80]
Merry et al, 2010 (Trained)	4.9%	0.00	[-0.80; 0.80]
Campa et al, 2020	8.0%	0.18	[-0.45; 0.80]
**Overall**	**29.8%**	**0.15**	**[-0.17; 0.47]**
Heterogeneity: $I^2 = 0\%$, $\tau^2 = 0$, $\chi^2_4 = 1.57$ ($p = 0.8144$)			
**Condition = Hot**			
Walsh et al, 1994	4.8%	-0.08	[-0.88; 0.73]
Gonzalez-Alonso et al, 1995	5.6%	0.14	[-0.60; 0.89]
Gonzalez-Alonso et al, 1997 (Hot)	3.0%	1.32	[0.31; 2.33]
Casa et al, 2000 (intravenous)	5.0%	0.78	[-0.01; 1.57]
Casa et al, 2000 (oral)	5.0%	0.78	[-0.01; 1.57]
Ebert et al, 2007	6.2%	-0.29	[-1.00; 0.42]
Del Coso et al, 2008	4.5%	0.71	[-0.12; 1.54]
Gagnon et al, 2012	12.7%	0.21	[-0.29; 0.70]
Kelly et al, 2012	7.8%	-0.27	[-0.90; 0.36]
James et al, 2017	5.7%	0.00	[-0.74; 0.74]
Funnell et al, 2019 (Blinded)	5.0%	0.53	[-0.26; 1.32]
Funnell et al, 2019 (Unblinded)	4.9%	0.56	[-0.24; 1.35]
**Overall**	**70.2%**	**0.30**	**[0.05; 0.55]**
Heterogeneity: $I^2 = 30.8\%$, $\tau^2 = 0.05$, $\chi^2_{11} = 15.9$ ($p = 0.1449$)			
**Overall**	**100.0%**	**0.24**	**[0.06; 0.42]**
Heterogeneity: $I^2 = 10.5\%$, $\tau^2 < 0.01$, $\chi^2_{16} = 17.88$ ($p = 0.3307$)			
Test for subgroup differences: $\chi^2_1 = 0.51$, df = 1 ($p = 0.4758$)			

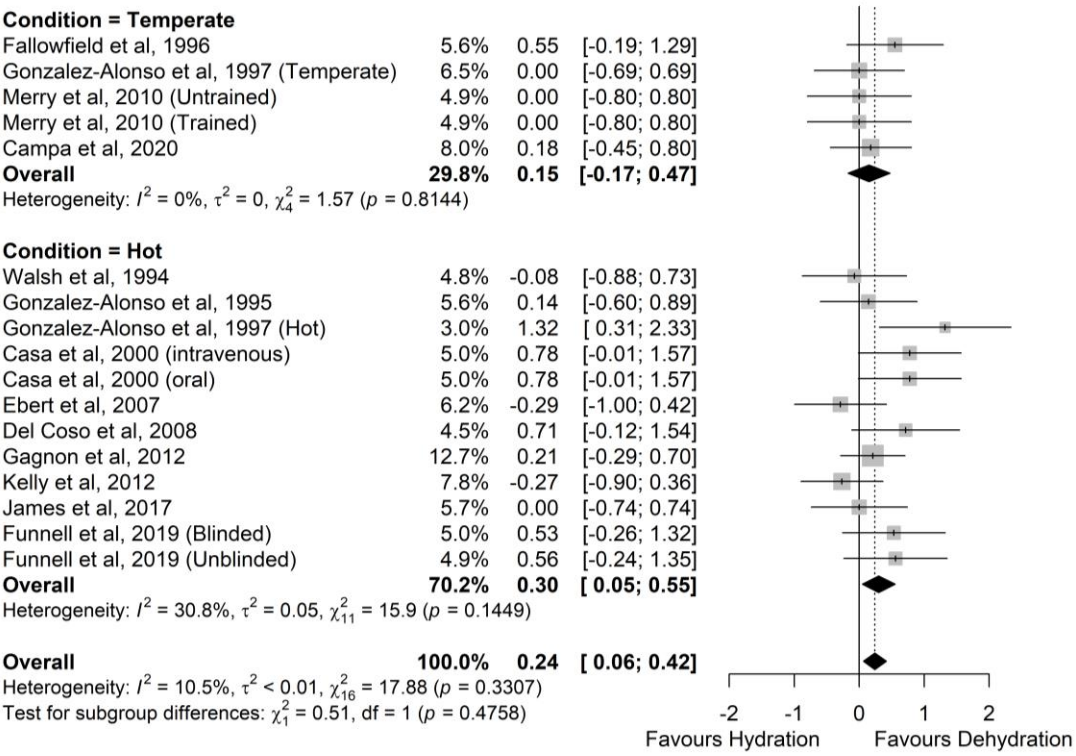

Figure 5. Comparison of the effects of prolonged exercise in hydrated *vs*. dehydrated status on carbohydrate oxidation. The upper part shows the studies performed in hot conditions (≥ 28°C); The lower part shows studies performed in temperate conditions. Forest plot shows standardised mean differences with 95% confidence intervals. Squares represent the standardised mean difference for each study. The diamond represents the pooled standardised mean difference for all studies. SMD standardised mean difference, CI confidence interval, df degrees of freedom.
