# Supplementary material for: The Effect of Heat Stress and Dehydration on Carbohydrate Use During Endurance Exercise: A Systematic Review and Meta-Analysis
Source: Sports Med. 2025 Aug 20;55(11):2825–47. doi: 10.1007/s40279-025-02294-3 (PMC12559103; doi:10.1007/s40279-025-02294-3)
Supplement: Supplementary file 4 — Supplementary file4 (PDF 280 KB) [file 40279_2025_2294_MOESM4_ESM.pdf]

Supplementary material 4: Meta-regressions

Title: The effect of heat stress and dehydration on carbohydrate use during endurance exercise: A systematic review and meta-analysis

Journal: Sports Medicine

Running heading: Heat stress and dehydration's impact on carbohydrate use in endurance exercise: A systematic review

Authors: Loïs Mougin[1], Heather Z Macrae[1], Lee Taylor[1], Lewis J James[1], Stephen A Mears[1*].

Affiliation(s):

[1] National Centre for Sport and Exercise Medicine, School of Sport, Exercise and Health Sciences, Loughborough University, Loughborough, United Kingdom.

***Corresponding author**

Stephen A Mears, School of Sport, Exercise and Health Sciences, National Centre for Sport and Exercise Medicine, Loughborough University, Loughborough, Leicestershire LE11 3TU, UK

Email: s.a.mears@lboro.ac.uk ; Phone: (+44) 1509 226391

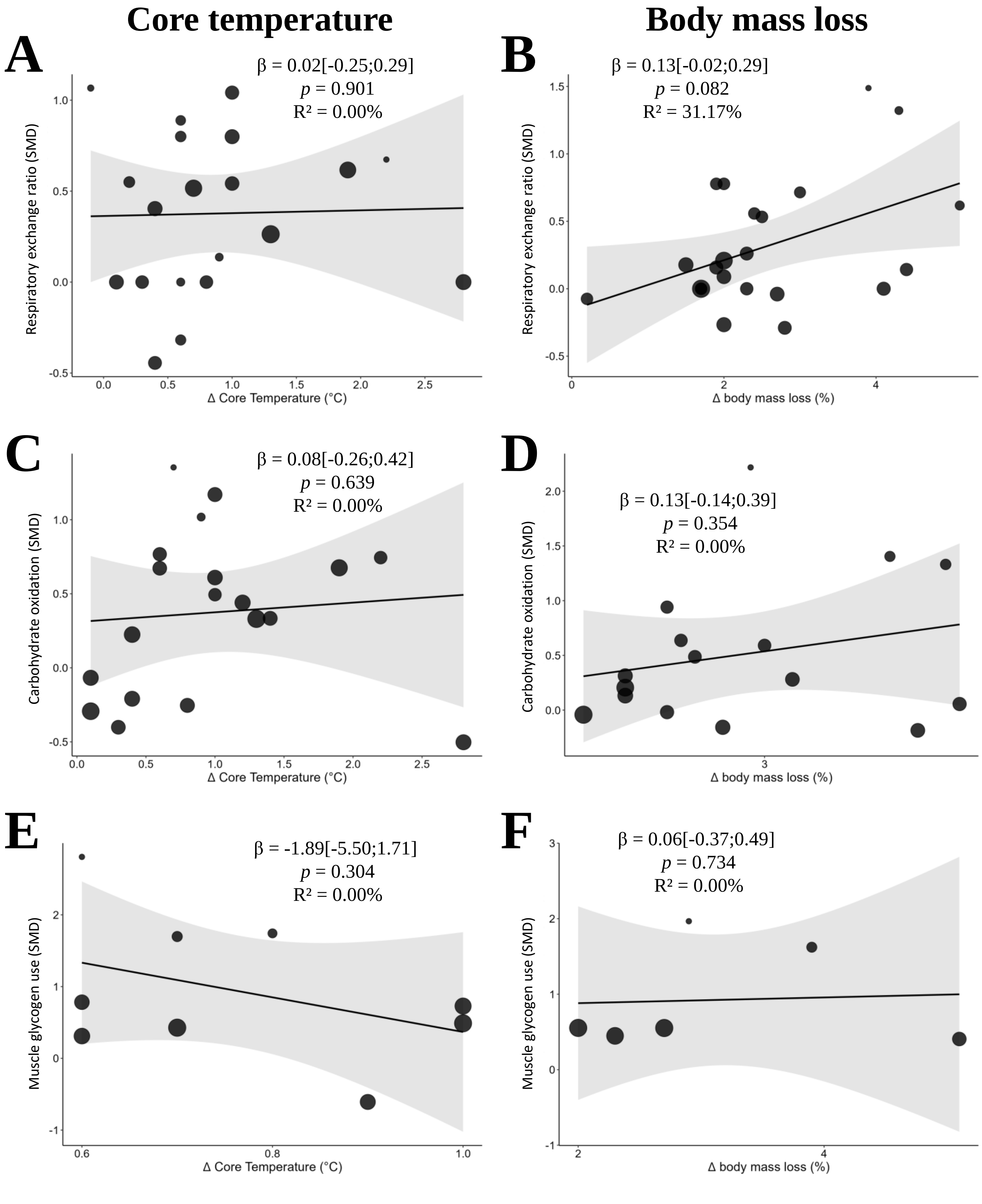

Figure 1. Meta-regression analyses examining the relationship between respiratory exchange ratio (A, B), carbohydrate oxidation (C, D), and muscle glycogen use (E, F) with changes in core temperature (left panel) and body mass loss (right panel). The left panel includes studies comparing hot vs. temperate conditions, while the right panel includes studies comparing hydrated vs. dehydrated states. Body mass loss is displayed inversely, where a value of 4 represents a −4% body mass loss in the dehydrated condition. β is the regression coefficient with 95% confidence interval. Each dot represents an individual study, with the size reflecting study precision.
